# Influences of Fluorine Substituents on Iminopyridine Fe(II)- and Co(II)-Catalyzed Isoprene Polymerization

**DOI:** 10.3390/polym10090934

**Published:** 2018-08-22

**Authors:** Guangqian Zhu, Xianhui Zhang, Mengmeng Zhao, Liang Wang, Chuyang Jing, Peng Wang, Xiaowu Wang, Qinggang Wang

**Affiliations:** 1 School of Chemical and Environmental Engineering, Shandong University of Science and Technology, Qingdao 266590, China; m18306422553@163.com; 2 Key Laboratory of Biobased Materials, Qingdao Institute of Bioenergy and Bioprocess Technology, Chinese Academy of Sciences, Qingdao 266101, China; zhangxh@qibebt.ac.cn (X.Z.); zhaomm@qibebt.ac.cn (M.Z.); wangliang@qibebt.ac.cn (L.W.); jingcy@qibebt.ac.cn (C.J.); 3 University of Chinese Academy of Sciences, Beijing 100049, China

**Keywords:** iminopyridine ligands, Fe(II), Co(II) complexes, isoprene polymerization, fluorinated aryl substituents

## Abstract

A series of iminopyridine complexes of Fe(II) and Co(II) complexes bearing fluorinated aryl substituents were synthesized for the polymerization of isoprene. The structures of complexes **3a**, **2b** and **3b** were determined by X-ray diffraction analysis. Complex **3a** contained two iminopyridine ligands coordinated to the iron metal center forming an octahedral geometry, whereas **2b** adopted a chloro-bridged dimer, and **3b** featured with two patterns of cobalt centers bridged via chlorine atoms. Complexes **2b** and **3b** represented rare examples of chlorine bridged bimetallic Co(II) complexes. The fluorine substituents effects, particularly on catalytic activity and polymer properties such as molecular weight and regio-/stereo-selectivity were investigated when these complexes were employed for isoprene polymerization. Among the Fe(II)/methylaluminoxane (MAO) systems, the 4-CF_3_ substituted iminopyridine Fe(II) complex **1a** was found as a highly active isoprene polymerization catalyst exhibiting the highest activity of 10^6^ g·(mol of Fe)^−1^·h^−1^. The resultant polymer displayed lower molecular weight (*M*_n_ = 3.5 × 10^4^ g/mol) and moderate polydispersity index (PDI = 2.1). Furthermore, the ratio of *cis*-1,4-/3,4 was not affected by the F substituents. In the series of Co(II)/AlEt_2_Cl binary systems, complexes containing electron-withdrawing N-aryl substituents (R = 4-CF_3_, 2,6-2F) afforded higher molecular weights polyisoprene than that was obtained by the complex containing electron-donating N-alkyl substituents (R = octyl). However, ternary components system, complex/MAO/[Ph_3_C][B(C_6_F_5_)_4_] resulted in low molecular weight polyisoprene (*M*_n_ < 2000) with high *trans*-1,4-unit (>95%).

## 1. Introduction

Synthetic polyisoprene displays significant interests in material application, which has been introduced to replace the natural rubber in some extent [1]. Polyisoprene can be obtained with diverse regio- and/or stereo-selectivities such as *cis*-1,4-/*trans*-1,4-/3,4-/1,2-polyisoprene. The physical properties are strongly related to the microstructure of polyisoprene. For instance, *cis*-1,4-polyisoprene shows similar characteristics particularly stereo and chemical compositions and thereby mechanical properties with the natural rubber which has been largely applied to tires [2]. High *trans*-1,4-polyisoprene is more alike to the natural gutta-percha [3], which is widely employed in shape memory functional materials. Furthermore, 3,4-polyisoprene is applied in wet skid resistance or low-rolling resistance tread due to its pendant groups [4]. Polyisoprene is generally produced via anionic or coordination polymerization [3,5]. In coordination polymerization, transition metal catalyzed polymerization has received considerable attention both from industry and academia because of their high reactivity and selectivity. In the past decades, various selective diene polymerization catalysts were investigated using Ziegler-Natta Ti [6] or rare earth metal catalysts [3,7,8,9,10,11,12,13]. Since the discovery of late transition metal catalysts for olefin polymerization reported by Gibson [14] and Brookhart [15] in the late 1990s, a plethora of catalysts have been developed for the polymerization of various type of monomers. Due to the lower Lewis acidic nature and higher functional group tolerance, late transition metal, especially cheap and earth-abundant iron- and cobalt-based catalysts, have attracted extensive investigations in both α-olefin and diene polymerization [16,17,18,19,20,21,22,23,24,25,26].

Despite iron- and cobalt-based catalysts in combination with alkylaluminium compounds for diene polymerization have been known for a long time, these structurally less-defined and multi-site catalysts often display low activity and produce polymers with poor stereoselectivity and broad molecular weight distribution, which results in poor mechanical properties. In order to gain better control of isoprene polymerization, including molecular weight, molecular weight distribution and stereoselectivity, academic and industrial researches have focused on well-defined organometallic single-site catalysts. Very recently, inspired by the Fe(II) bisiminopyridine complexes in ethylene polymerization, Ritter group [27] reported a ternary catalyst system, [iminopyridine Fe(II) complexes, alkyl aluminium and dealkylating reagent [Ph_3_C][B(C_6_F_5_)_4_]] that showed high stereoselectivity for isoprene polymerization. The iron catalysts bearing electron donating imine groups afforded polymer with high *trans*-1,4 selectivity, while high *cis*-1,4 selectivity was obtained using the catalysts bearing electron-withdrawing imine group. In view of these findings, the authors proposed that higher electron density at the iron center favor *trans*-1,4 selectivity. Later, Chen et al. [28] reported Fe(II) and Co(II) complexes bearing bulky substituents. The complexes containing N-aryl imine groups produced polymer in a higher yield than N-alkyl imine groups, which potentially suggests that electron-withdrawing substituents lead to better monomer coordination and faster chain propagation.

**Scheme 1:**
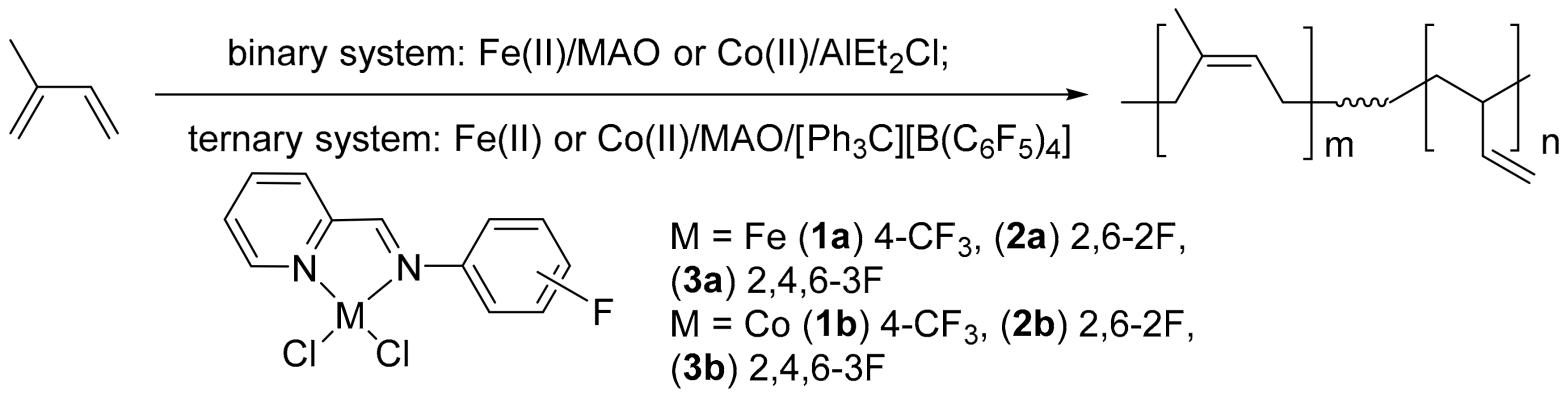
Fluorinated-aryl-substituted iminopyridine Fe(II) and Co(II) complexes catalyzed isoprene polymerization.

In the present study, a series of iminopyridine Fe(II) and Co(II) complexes containing fluorinated aryl substituents were synthesized and employed in isoprene polymerization in combination with alkylaluminium (methylaluminoxane (MAO) or AlEt_2_Cl) (Scheme 1). Both binary system and ternary system were investigated. The fluorine effects of the imine moiety on the catalytic activity, molecular weight as well as the regio- and/or stereoselectivity were investigated. The present study sheds light on how to control the isoprene polymerization through modifying the ligand framework and electronic effects of the Fe(II) and Co(II) complexes catalysts.

## 2. Materials and Methods

### 2.1. General Information

All manipulations of air and/or moisture sensitive compounds were performed using standard Schlenk technique. Toluene, dichloromethane, tetrahydrofuran (THF) and hexane were purchased from Sinopharm Chemical Reagent (Shanghai, China), dried over sodium benzophenone ketyl (toluene, THF) or calcium hydride (CH_2_Cl_2_, hexane) and distilled under an argon atmosphere prior to use. The progress of the organic reactions was monitored by thin layer chromatography (TLC) using 0.2 mm Macherey-Nagel silica gel precoated plates (POLYGRAM SILG/UV254, haoranbio, Shanghai, China). ^1^H and ^13^C NMR spectra were recorded on a Bruker Avance III 400 MHz spectrometer (Bruker, Karlsruhe, Germany) using CDCl_3_ (CIL, Andover, MA, USA) as solvent and trimethylsilane (TMS; CIL, Andover, MA, USA) as internal reference. Chemical shifts and coupling constant are given in ppm and in Hz respectively. The abbreviations: s = singlet, d = doublet, t = triplet, m = multiplet were used to designate the multiplicities of signal. Attenuated total reflection-infrared (ATR-IR) spectroscopy was performed using Thermo Scientific Nicolet iN10 (Thermo Fisher Scientific Corporation, Waltham, MA, USA). Elemental analysis was carried out on Vario EL III elemental analyzer (Elementar Corporation, Hanau, Germany) at Shanghai Institute of Organic Chemistry, Chinese Academy of Sciences (Shanghai, China). Mass spectra for new organic compounds were measured using maXis II of Bruker Daltonics Corporation (Bruker Daltonics Corporation, Billerica, MA, USA), while for Fe(II) and Co(II) complexes were recorded by ACQUITYTM UPLC & Q-TOF MS Premier (Waters, Milford, MA, USA) at Analytical Center of Shanghai Jiao Tong University (Shanghai, China). X-ray Diffraction data was collected on Smart 1000 diffractometer (Bruker, Karlsruhe, Germany) with Mo K-alpha X-ray source (λ = 0.71073 Å) at 150 K. The SUPERFLIP72 program was employed for dealing with the collected data and refinement of data was achieved through the SHELX-97 and OLEX2 programs. Molecular weights and polydispersity index (PDI) of polyisoprene were undertaken by gel permeation chromatography (GPC, Viscotek VE2001 GPC, Viscotek Corporation, Houston, TX, USA) at 35 °C using a PL2MB500A column in THF at a flow rate of 1 cm^3^·min^−1^; 50 µL was injected using a Viscotek VE2001 GPC equipped with a Viscotek VE3580 Refractive Index detector using THF as the eluent. While high temperature gel permeation chromatography (HGPC, PL-GPC 220, Agilent Technologies, Palo Alto, CA, USA) was employed for polymerization using trichlorobenzene as the eluent and polystyrenes as standards. Isoprene was purchased from Aladdin Industrial Corporation (Shanghai, China), dried over CaH_2_ and distilled prior to use. 1-(pyridin-2-yl)-*N*-(4-(trifluoromethyl)phenyl)methanimine (**L1**), (*E*)-*N*-(2,6-difluorophenyl)-1-(pyridin-2-yl)methanimine (**L2**), (*E*)-2,4,4-Trimethyl-*N*-(pyridin-2-ylmethylene)pentan-2-amine (**L4**) and 1-(pyridin-2-yl)-*N*-(2,4,6-(triphenyl)phenyl)methanimine (**L5**) are not commercially available and can be prepared using reported procedure [29,30,31,32,33]. Complexes **4a** (C_14_H_22_Cl_2_FeN_2_), **5a** (C_60_H_44_Cl_4_Fe_2_N_4_) and **4b** (C_14_H_22_Cl_2_CoN_2_), **5b** (C_30_H_22_Cl_2_CoN_2_) were synthesized using reported routes [27,28]. All other reagents were purchased from commercial sources and used without further purification. Due to the paramagnetic properties of Fe(II) and Co(II) complexes, these complexes were mainly characterized by elemental analyses, TOF-MS-ES+ (the concrete graphical data see Supplementary Materials, Figures S5–S10) and Attenuated total reflection-infrared spectroscopy (ATR-IR) unless otherwise noted.

### 2.2. Experimental Methods

#### 2.2.1. Synthesis of 1-(Pyridin-2-yl)-*N*-(2,4,6-trifluorophenyl)methanimine (**L3**)

Note: The **L1**, **L2**, **L4** and **L5** are known compounds and can be prepared using reported procedure. However, the procedure adopted for the synthesis of **L3** is slightly different in the context of purification process and can be prepared in the following procedure. To a solution of 2-pyridinecarboxaldehyde (1.00 g, 0.89 mL, 6.80 mmol) in CH_2_Cl_2_ (10 mL), 2,4,6-trifluoroaniline (0.72 g, 6.73 mmol) was added. The reaction mixture was heated to reflux for 12 h with azeotropic removal of water using a Dean-Stark trap. On completion of the reaction (checked by TLC), the reaction mixture concentrated under reduced pressure and the residue was purified by recrystallization using hexane at 0 °C afforded **L3** as colorless solid (0.81 g, 50.5% yield).

NMR Spectroscopy: ^1^H NMR (400 MHz, CDCl_3_, 298 K), δ: 8.75 (s, 1H), 8.72–8.71 (m, 1H), 8.25 (d, *J* = 7.8 Hz, 1H), 7.85–7.80 (m, 1H), 7.41–7.38 (m, 1H), 6.79–6.74 (m, 2H) ppm. ^13^C{^1^H} NMR (100 MHz, CDCl_3_, 298 K), δ: 167.3–167.2 (m), 161.1–160.8 (t), 158.6–158.3 (t), 156.9–156.7 (m), 154.4–154.2 (m), 149.9 (s), 136.8 (s), 125.9 (s), 121.9 (s), 101.2–100.6 (m) ppm. ^19^F NMR (376 MHz, CDCl_3_, 298 K), δ: −111.31–111.38 (m, 1F), −119.98–120.01 (m, 2F) ppm. HRMS-ESI (*m*/*z*): Calcd for [C_12_H_7_F_3_N_2_ + H], 237.0634. Found, 237.0631; Calcd. for [C_12_H_7_F_3_N_2_ + Na], 259.0454. Found, 259.0454.

#### 2.2.2. Synthesis of Iminopyridine Fe(II) Complexes **1a**, **2a**, **3a**

##### Synthesis of **1a**

In a glovebox, a solution of ligand **L1** (125.0 mg, 0.50 mmol) in CH_2_Cl_2_ (8 mL) was added to anhydrous FeCl_2_ (63.3 mg, 0.50 mmol) in 25 mL Schlenk flask. The mixture was stirred for 48 h at room temperature. The precipitate was collected by filtration in argon atmosphere, washed with distilled hexane (10 mL × 2) and dried under vacuum to obtain gray solid **1a** (112.6 mg, 59.8% yield). TOF-MS-ES+ (*m*/*z*): calcd. for C_26_H_18_ClF_6_FeN_4_: 591.0474, found: 591.0487 [M − Cl]^+^. Anal. calcd. for C_13_H_9_Cl_2_F_3_FeN_2_: C, 41.43; H, 2.41; N, 7.43; found: C, 41.41; H, 2.65; N, 7.11. ATR-IR (cm^−1^): 3070, 1592 ν(C=N), 1446, 1322, 1160, 1125, 1069, 856, 828, 786.

##### Synthesis of **2a**

In a glovebox, a solution of ligand **L2** (150.0 mg, 0.69 mmol) in CH_2_Cl_2_ (8 mL) was added to anhydrous FeCl_2_ (87.1 mg, 0.69 mmol) in 25 mL Schlenk flask. The reaction was stirred for 48 h at room temperature. The precipitate was collected by filtration in argon atmosphere, washed with distilled hexane (10 mL × 2) and dried under vacuum to obtain atropurpureus solid **2a** (168.1 mg, 73.0% yield). TOF-MS-ES+ (*m*/*z*): calcd. for C_24_H_16_ClF_4_FeN_4_: 527.0350, found: 527.0359 [M − Cl]^+^. ATR-IR (cm^−1^): 3044, 1639, 1598 ν(C=N), 1471, 1304, 960, 809, 779. Note: We have performed the elemental analysis of **2a**, the obtained value is beyond the requirement of publishable results. Anal. calcd. for C_1__2_H_8_Cl_2_F_2_FeN_2_: C, 41.78; H, 2.34; N, 8.12; found: C, 39.72; H, 3.51; N, 7.47.

##### Synthesis of **3a**

In a glovebox, a solution of ligand **L3** (200.0 mg, 0.85 mmol) in CH_2_Cl_2_ (8 mL) was added to anhydrous FeCl_2_ (120.1 mg, 0.85 mmol) in 25 mL Schlenk flask. The reaction mixture was stirred for 48 h at room temperature. The precipitate was collected by filtration in argon atmosphere, washed with distilled hexane (10 mL × 2) and dried under vacuum to obtain purple solid **3a** (96.2 mg, 31.3% yield). TOF-MS-ES+ (*m*/*z*): calcd. for C_24_H_14_ClF_6_FeN_4_: 563.0161, found: 563.0170 [M − Cl]^+^. ATR-IR (cm^−1^): 3034, 1626, 1598 ν(C=N), 1502, 1455, 1446, 1121, 1044, 836, 819, 778. Note: We have performed the elemental analysis of **3a**, the obtained value is beyond the requirement of publishable results. Anal. calcd. for C_12_H_7_Cl_2_F_3_FeN_2_: C, 39.71; H, 1.94; N, 7.72; found: C, 38.47; H, 2.23; N, 7.42. Single crystal suitable for X-ray diffraction analysis was obtained from concentrated acetonitrile solutions of complex **3a** layered with hexane at −35 °C.

#### 2.2.3. Synthesis of Iminopyridine Co(II) Complexes **1b**, **2b**, **3b**

##### Synthesis of **1b**

In a glovebox, a solution of ligand **L1** (125.0 mg, 0.48 mmol) in THF (8 mL) was added to anhydrous CoCl_2_ (64.9 mg, 0.48 mmol) in 25 mL Schlenk flask. The reaction was stirred for 48 h at room temperature. The precipitate was collected by filtration in argon atmosphere, washed with distilled hexane (10 mL × 2) and dried under vacuum to obtain green solid **1b** (141.1 mg, 74.3% yield). TOF-MS-ES+ (*m*/*z*): calcd. for C_26_H_18_ClCoF_6_N_4_: 594.0456, found: 594.0463 [M − Cl]^+^. Anal. calcd. for C_13_H_9_Cl_2_F_3_CoN_2_: C, 41.08; H, 2.39; N, 7.37; found: C, 41.87; H, 2.84 N, 7.98. ATR-IR (cm^−1^): 3066, 1611, 1597 ν(C=N), 1323, 1166, 1123, 1066, 847, 778.

##### Synthesis of **2b**

In a glovebox, a solution of ligand **L2** (150.0 mg, 0.69 mmol) in THF (8 mL) was added to anhydrous CoCl_2_ (89.3 mg, 0.69 mmol) in 25 mL Schlenk flask. The reaction was stirred for 48 h at room temperature. The precipitate was collected by filtration in argon atmosphere, washed with distilled hexane (10 mL × 2) and dried under vacuum to obtain blackish green solid **2b** (216.0 mg, 90.3% yield). TOF-MS-ES+ (*m*/*z*): calcd. for C_24_H_16_ClCoF_4_N_4_: 530.0331, found: 530.0326 [M − Cl]^+^. Anal. calcd. for C_12_H_8_Cl_2_F_2_CoN_2_: C, 41.41; H, 2.32; N, 8.05; found: C, 41.71; H, 2.28; N, 7.51. ATR-IR (cm^−1^): 3072, 1637, 1599 ν(C=N), 1472, 1239, 1013, 814, 776. Single crystal suitable for X-ray diffraction analysis was obtained from concentrated acetonitrile solutions of complex **2b** layered with hexane at −35 °C.

##### Synthesis of **3b**

In a glovebox, a solution of ligand **L3** (150.0 mg, 0.64 mmol) in THF (8 mL) was added to anhydrous CoCl_2_ (82.5 mg, 0.64 mmol) in 25 mL Schlenk flask. The reaction was stirred for 48 h at room temperature. The precipitate was collected by filtration in argon atmosphere, washed with distilled hexane (10 mL × 2) and dried under vacuum to obtain emerald green solid **3b** (190.1 mg, 81.8% yield). TOF-MS-ES+ (*m*/*z*): calcd. for C_24_H_14_Cl_3_Co_2_F_6_N_4_: 694.8853, found: 694.8864 [M − Cl]^+^. Anal. calcd. for C_12_H_7_Cl_2_F_3_CoN_2_: C, 39.38; H, 1.93; N, 7.65; found: C, 39.87; H, 2.46; N, 6.88. ATR-IR (cm^−1^): 3067, 1637, 1598 ν(C=N), 1500, 1452, 1124, 1047, 999, 850, 778. Single crystal suitable for X-ray diffraction analysis was obtained from concentrated acetonitrile solutions of complex **3b** layered with hexane at −35 °C.

#### 2.2.4. General Procedure for Isoprene Polymerization

In a typical experiment, the reactor was heated, dried in a vacuum, and recharged with nitrogen for three times. When the reactor cooled down, an aluminum cocatalyst, toluene (5 mL), isoprene (2 mL, 20 mmol) and a solution of Fe(II) or Co(II) complexes (8 μmol) in 1 mL CH_2_Cl_2_ added into the reactor in sequence manner. The reaction was employed for 2 h and was then quenched with a diluted HCl solution of methanol (MeOH/HCl = 50/1). The polymer was collected by filtration and washed with ethanol several times and dried at room temperature for 24 h under vacuum.

For ternary system, a slightly different method of polymerization was used. A 25 mL Schlenk reactor was heated, dried in a vacuum, and recharged with nitrogen for three times. On cooling the reactor, Fe(II) or Co(II) complexes (8 μmol) in 2 mL toluene, MAO (40 μmol) in 1 mL toluene were added. The reaction mixture was stirred for 2 min and [Ph_3_C][B(C_6_F_5_)_4_] (7.4 mg, 8 μmol) was added as a solution in toluene (2 mL). The reaction mixture was stirred for 2 min and isoprene (1 mL, 10 mmol) was added and stirred for 2 h at room temperature. The polymerization was quenched with a diluted HCl solution of methanol (MeOH/HCl = 50/1). The polymer was collected by filtration and washed with ethanol several times and dried at room temperature for 24 h under vacuum.

## 3. Results and Discussion

### 3.1. The Synthesis and Characterization of Iminopyridine Ligands and Corresponding Fe(II) and Co(II) Complexes

**Scheme 2:**
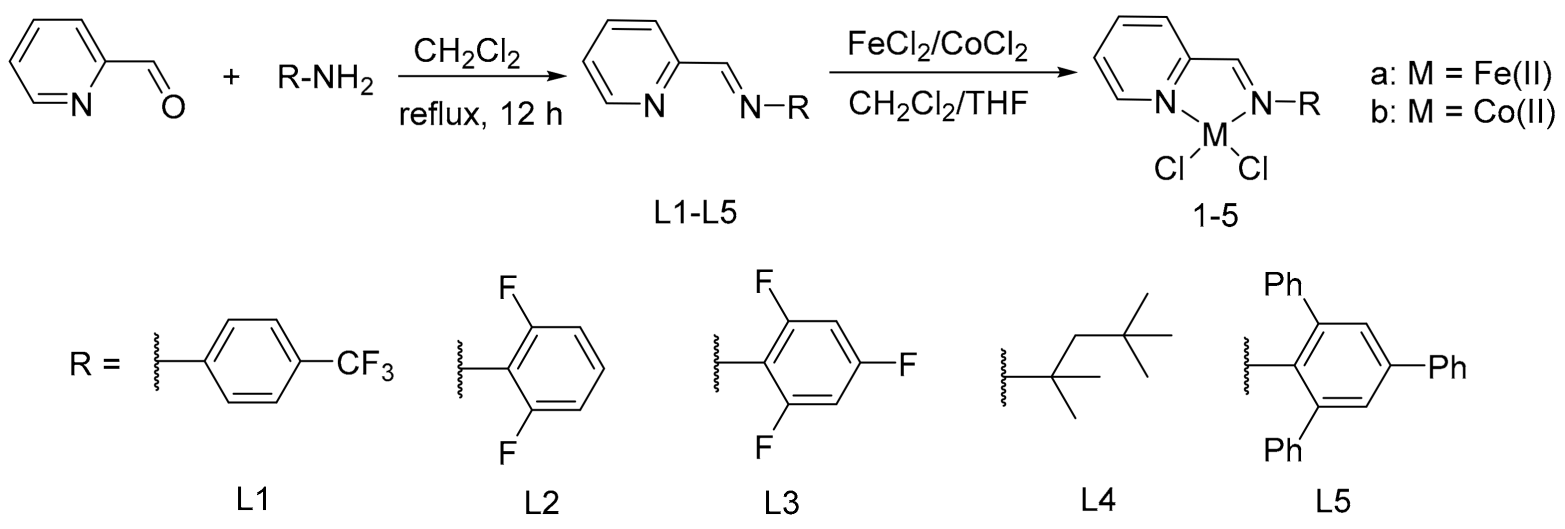
Synthesis of iminopyridine free ligands and the Fe(II) and Co(II) complexes.

Ligand precursors were prepared by the condensation reaction of 2-pyridinecarboxaldehyde with arylamine in dichloromethane at refluxed temperature (Scheme 2). The desired compounds were purified through recrystallization in hexane (**L1**, **L3**), reduced pressure distillation (**L4**) or column chromatography (**L2**, **L5**). Ligands **L1**, **L2**, **L4**, and **L5** were confirmed according to the references [29,30,31,32,33] and novel ligand **L3** was characterized by ^1^H NMR, ^13^C NMR, ^19^F NMR, and HRMS-ESI (See Supplementary Materials, Figures S1–S4).

The corresponding iminopyridine Fe(II) and Co(II) dichloride complexes containing fluorine substituents were synthesized by a reaction of the resulting iminopyridine ligand with one equivalent of anhydrous FeCl_2_ in CH_2_Cl_2_ or anhydrous CoCl_2_ in THF, respectively (Scheme 2). These complexes were characterized by ATR-IR, TOF-MS-ES+- and elemental analysis.

**Figure 1:**
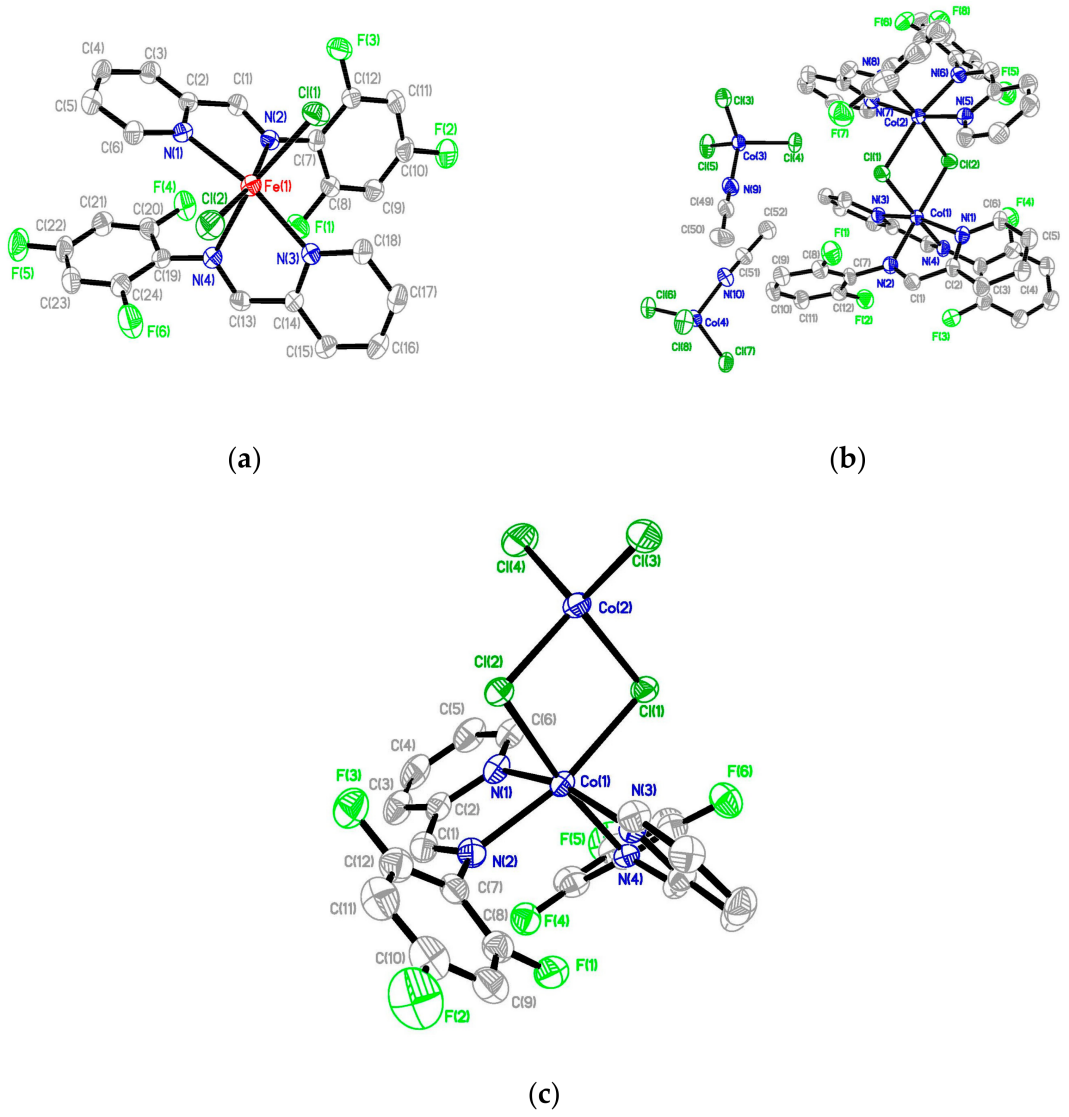
(**a**) ORTEP-type view of the structure of complex **3a**, drawn at 30% of probability. Hydrogen atoms and CH_3_CN molecule have been omitted for clarity; (**b**) ORTEP-type view of the structure of complex **2b**, drawn at 30% of probability. Hydrogen atoms and CH_3_CN molecule have been omitted for clarity; (**c**) ORTEP-type view of the structure of complex **3b**, drawn at 30% of probability. Hydrogen atoms and CH_3_CN molecule have been omitted for clarity.

The structures of complexes **3a**, **2b**, and **3b** were further characterized by X-ray crystallographic analysis. The ORTEP drawings of Fe(II) complex **3a** and Co(II) complexes **2b** and **3b** are shown in Figure 1 (Concrete crystallographic data see Supplementary Materials, Tables S7–S10). For these complexes, the ligands system adopts a cisoid conformation, which permits both pyridine nitrogen atoms and imine nitrogen atoms to coordinate to the metal atoms. Single crystal of Fe(II) complex **3a** (See Figure 1a) was obtained by recrystallization from concentrated acetonitrile solutions of complex **3a** layered with hexane at −35 °C. The solid state crystal structure analysis shows that the complex **3a** adopts distorted octahedral coordination geometry around the iron metal formed by two iminopyridine ligands and two terminal chlorine atoms. And the imine nitrogen atom (N2) and Cl2 occupy the axial coordination sites and the axial N2–Fe1–Cl2 angle exhibits a small non-linearity (167.46(12)°). The bond distances of Fe–N(iminoaryl) [Fe1–N2: 2.304(5) Å, Fe1–N4: 2.322(5) Å] and Fe–N(pyridyl) [Fe1–N1: 2.204(5) Å, Fe1–N3: 2.215(5) Å)] are all longer than the corresponding Fe–N bond distances [Fe1–N2A(iminoalkyl): 2.121(6) Å, Fe1–N1A(pyridyl): 2.119(5) Å)] in Ritter’s complex (CCDC number: 853130) [27], which may be supported that electron-withdrawing substituent (2,4,6-3F group in **3a**) reduces the electron density around the metal center and affects binding interaction between the metal and the coordination donor as well as the opening environment near the metal center. Complex **3a** features a smaller N1–Fe1–N2 bond angle of 72.68(17)°, N3–Fe1–N4 bond angle of 72.58(17)° and Cl1–Fe1–Cl2 bond angle of 98.24(7)° than N1A–Fe1–N2A bond angle of 78.3(2)° and Cl1A–Fe1–Cl2A bond angle of 121.77(8)° in Ritter’s complex. These imply congestion around the Fe center. The Fe–Cl bond lengths are about 2.396(19)–2.421(18) Å [Fe1–Cl1 2.4207(18) Å; Fe1–Cl2 2.3958(19) Å], which is longer than the Ritter’s complex [Fe1–Cl1A 2.238(19) Å; Fe1–Cl2A 2.244(2) Å].

Single crystals of Co(II) complexes **2b** and **3b** were obtained by recrystallization from concentrated acetonitrile solutions of complexes layered with hexane at −35 °C. **2b** and **3b** represented rare examples of chlorine bridged bimetallic cobalt complexes, which led to more distorted angles (average angles: 70.63° for **2b** and 70.83° for **3b**) between the planes of aryl substituents at the imine nitrogen atom and the pyridine moiety than similar iminopyridine Co(II) complex (angle between the planes: 89.84°) as reported in the previous report [34]. Complex **2b** (See Figure 1b) exists as an ion pair, [L_2_CoCl_2_][CoCl_3_(CH_3_CN)]_2_ with cationic cobalt atom coordinated with two ligands and another two cobalt atoms each with three chlorine atoms and one acetonitrile molecule, respectively. The cation shows a chloride-bridged centrosymmetric dinuclear structure and two equivalent iminopyridine ligands coordinated with one cobalt atom. Each Co(II) ion is in a distorted octahedral geometry with the equatorial plane occupied by the two pyridine N atoms, one imine N atom and one bridging chlorine atom. The axial positions are occupied by the other imine N atom and the other bridging chlorine atom. The Cl1–Co1–Cl2 bond angle (86.71(11)°) is similar to the Cl1–Co2–Cl2 bond angle (86.32(11)°), and in the cation of complex **2b**, Co–N bond distances and Co–Cl bond distances are almost the same for both cobalt metal centers. An ion-paired complex [L_2_Co]^2+^[CoCl_4_]^2−^ (A) was reported by D. Gong et al. in which L denotes tridentate N,N,N ligands [35]. In complex **2b**, Co–N (pyridyl) bond distances range from 2.102(11) to 2.128(10) Å [Co1–N1 2.128(10) Å; Co1–N3 2.121(10) Å; Co2–N5 2.102(11) Å; Co2–N7 2.120(10) Å], which are slightly longer than the typical Co–N(pyridyl) bond distances of reported Co(II) complexes in the range of 2.032–2.035 Å. Co–N(iminoaryl) bond distances are about 2.140(10)–2.178(10) Å [Co1–N2 2.140(10) Å; Co1–N4 2.171(9) Å; Co2–N6 2.149(10) Å; Co2–N8 2.178(10) Å], while those of Co–N(iminoaryl) are between 2.191–2.304 Å. The Co–Cl bond distances in the cation of **2b** are about 2.429(3)–2.453(3) Å [Co1–Cl1 2.429(3) Å; Co1–Cl2 2.442(3) Å; Co2–Cl1 2.453(3) Å; Co2–Cl2 2.435(3) Å]. The Co–Cl bond distances are comparable to those of reported bisiminopyridine ligated Co(II) complexes with bond distances ranging from 2.244–2.284 Å [36,37]. The counter anions consist of two separated CH_3_CN·CoCl_3_, in which cobalt displays distorted tetrahedral geometry. Interestingly, there is a big difference in the bond distances of C≡N coordinated to the Co atoms. In comparison with the bond distances of C≡N triple bond of free CH_3_CN (C≡N 1.160 Å) [38], the bond distances of CH_3_CN coordinated to Co3 is shortened (N9–C49 1.115(18) Å), while the bond distances of CH_3_CN coordinated to Co4 is almost intact (N10–C51 1.150(18) Å). There are few examples of structurally characterized cobalt complexes containing one or two XCoCl_3_ fragments (X = Cl, S). Recent publication from Braunstein and coworkers reported dinuclear cobalt complexes contained one CoCl moiety ligated with two ligands and one CoCl_3_ fragment coordinated by S atom of the ligand (Figure S44) [39]. In the anionic fragment, the Co center (Co2) adopts a distorted tetrahedral coordination geometry with three similarly coordinated Cl ligands [Co−Cl bond lengths are in the range 2.236(2)−2.246(2) Å], which are slightly longer than the Co–Cl bond distances in the anion of **2b** [Co3–Cl3 2.240(4) Å; Co3–Cl4 2.246(4) Å; Co3–Cl5 2.234(4) Å; Co4–Cl6 2.237(4) Å; Co4–Cl7 2.254(5) Å; Co4–Cl8 2.219(5) Å]. The Co2−S2 bond of 2.396(2) Å is significantly longer than the Co–N(acetonitrile) bond distances [Co3–N9 2.053(13) Å; Co4–N10 2.031(13) Å]. Meanwhile, they presented structurally characterized zwitterionic cobalt complexes with anionic cobalt(II) centers in the form of [L]CoCl_3_ and [L]_2_CoCl_4_ (Figure S44). The anionic Co(II) center of [L]CoCl_3_ displays a distorted tetrahedral environment with Co–Cl bond distances ranging from 2.233(8) (Cl2) to 2.264(8) (Cl3) Å, slightly longer to those found in **2b** [2.219(5)–2.246(4) Å]. Interestingly, The Co–Cl bond distances of anionic Co(II) center of [L]_2_CoCl_4_ range from 2.257(6)–2.293(5) Å, which are much longer than those found in **2b**. The angles involving N of acetonitrile are in the range of 102.7(4)° [N9–Co3–Cl3] to 109.6(4)° [N9–Co3–Cl5], which are broader than those involving only chloride and the cobalt center [between Cl5–Co3–Cl4 111.92(17)° and Cl5–Co3–Cl3 113.70(18)°]. Compared with complex **3b**, the replacement of chlorines by two ligands on the second cobalt metal center can be explained that two terminal chlorines are displaced by the less electron-withdrawing ligand **L2**, the leaving Cl anion is captured by CoCl_2_ and CH_3_CN, existed in the form of CH_3_CN·CoCl_3_.

Complex **3b** (See Figure 1c) features a chloride-bridged dinuclear structure as one cobalt ion is in a distorted octahedral coordination geometry while the other is in a distorted tetrahedral environment. In Co1 metal center, two equivalent iminopyridine ligands coordinated with it, the equatorial plane is occupied by pyridine nitrogen atom (N1, N3), imine nitrogen atom N2 and Cl1. The axial positions are coordinated by the imine nitrogen atom N4 and Cl2, which exhibits a small non-linearity [the N4–Co1–Cl2 bond angle is 171.61(13)°]. The N1–Co1–N2 bond angle is 77.4(2)°, and the N3–Co1–N4 bond angle is 76.76(18)°, which leads to a big block in Co1 metal center. The Co1–Cl bond lengths are 2.4647(17) Å (Co1–Cl1) and 2.4881(16) Å (Co1–Cl2) and Cl1–Co1–Cl2 bond angle is 87.07(5)°. The central metal atom Co2 can be best described as distorted tetrahedral coordination geometry with Cl2–Co2–Cl1 angle of 94.32(6)° and Cl4–Co2–Cl3 angle of 112.60(9)°. The Co2–Cl1 and Co2–Cl2 bond lengths [2.3289(16) Å and 2.3235(18) Å] are shorter than the Co1–Cl1 and Co1–Cl2 bond lengths [2.4647(17) Å and 2.4881(16) Å] while longer than the Co2–Cl3 and Co2–Cl4 [2.2302(18) Å and 2.209(2) Å]. The imino C–N bond distances [N2–C1 1.296(8) Å, N4–C13 1.267(7) Å] are consistent with the typical characters of C=N double bond although it is relatively shorter than those in the literature [40,41,42]. The Co1–Co2 distance of 3.367(8) Å is longer than those found in some dicobalt complexes and carbonyl cobalt compounds [43,44].

### 3.2. Isoprene Polymerization Studies

#### 3.2.1. Polymerization of Isoprene with Fe(II) Catalysts

A variety of alkylaluminum reagents (MAO, Al(*i*-Bu)_3_, AlEt_3_, AlEt_2_Cl, AlEtCl_2_, and EASC (Ethylaluminum sesquichloride)) were screened as a cocatalyst in isoprene polymerization (See Supplementary Materials, Table S1) and MAO was chosen as the effective cocatalyst. The polymerization results are summarized in Table 1 (NMR Spectra and GPC characterizations of the representative polyisoprene see Supplementary Materials, Figures S11–S16 and S25–S29).

The fluorinated aryl moiety significantly influenced the catalytic performances of the complexes. The fluorinated aryl-substituted complexes **1a**–**3a** produced polymers at higher yields (32.7–99.0%) than the reported complexes **4a**–**5a** (10.9–21.1%) under the same condition. In Fe(II)/MAO binary system, complex **1a** bearing 4-CF_3_ moiety exhibited the highest yield but with a relatively lower molecular weight and broad molecular weight distribution (Table 1, entry 1). Complex **2a** having 2,6-2F moiety gave slightly lower activity with a higher molecular weight and narrow molecular weight distribution (Table 1, entry 2). In contrast, complex **3a** with 2,4,6-3F showed the lowest activity with a higher molecular weight and narrower molecular weight distribution (Table 1, entry 3). It is proposed that introducing electron withdrawing group can reduce the electron density on the metal center and the increased Lewis acidic character facilitates the coordination of isoprene monomer, leading to the increment of chain propagation rate. The lower molecular weight of polyisoprene generated with complex **1a** may be ascribed to a faster chain transfer process. Furthermore, in Fe(II)/MAO system, the ratio between *cis*-1,4-/3,4-units was not affected by the F substituents. Although this difference is not fully understood, it is clear that the cocatalyst may play an important role in determining the stereoselectivities.

Subsequently, complex **1a** was used as a model catalyst to study the Al/M ratio and catalyst loading influence on the isoprene polymerization. Gratifyingly, complex **1a** can polymerize isoprene in full conversion within 10 min under our typical condition (See Supplementary Materials, Table S2, entry 1). Because of the poor solubility of the resultant polyisoprene, the molecular weight and molecular weight distribution cannot be obtained via GPC measurement. Decreasing the Al/M ratio from to 500 to 10 resulted in a steady decline of obtained polymer yield but with a narrow PDI and an increase of the molecular weight. At Al/M = 500, the stereoselectivity of the resultant polyisoprene was *cis*-1,4-/3,4- = 54/46. Decreasing the Al/M ratio and catalyst loading or increasing the Al/M ratio with lower catalyst loading resulted in an inverse of the *cis*-1,4-/3,4- ratio (See Supplementary Materials, Table S2, entries 2, 3 and 6, 7; representative NMR spectra see Supplementary Materials, Figures S23 and S24). At very low Al/M ratio (Al/Fe = 10), prolonging reaction time did not increase the polymer yield, which indicated a rapid deactivation process (See Supplementary Materials, Table S2, entries 3–5).

At Al/M = 500, the catalyst loading of complex **1a** can even be reduced to 1 μmol to give reasonable yield of polyisoprene (See Supplementary Materials, Table S2, entry 6). Increasing reaction time resulted in little improvement on the polymer yield (See Supplementary Materials, Table S2, entries 8 and 9), which produced polyisoprene in 64.9% yield with a bimodal character. One active species generated high molecular weight of polyisoprene, the other generated low molecular weight of polyisoprene with narrow PDI. Prolonging the reaction time to 40 min and 1 h did not increase the yield of polyisoprene, which implied active species were rather sensitive and short-lived. However, based on the molecular weight of the obtained polyisoprene, it seems that the bimodal active species turned to unimodal species, which produced high molecular weight of polyisoprene along the reaction time [See Supplementary Materials, Table S2, entry 6: 9.3 × 10^4^/1700 (10 min), entry 8: 4.4 × 10^4^ (40 min), and entry 9: 21.1 × 10^4^ (1 h)]. Increasing the ratio of Al/Fe from 500 to 1000, polyisoprene is obtained in 69.0% yield (See Supplementary Materials, Table S2, entry 7). It was concluded that large excess of MAO did not enhance the polymerization activity and stereoselectivity, while molecular weight and PDI showed an increasing trend.

For complex **1a**, at very low catalyst loading (1 μmol), we observed an increase of polymer yield when Al/M ratio was increased from 500 to 1000 (See Supplementary Materials, Table S2, entries 6 and 7). Therefore, we systematically investigated higher Al/M ratio on all the complexes **2a**–**5a** (See Supplementary Materials, Table S3). The results showed that the polyisoprene yields increased with the increase of Al/Fe ratio while stereoselectivity changed little when novel fluoro-substituted complexes **2a** and **3a** were employed. The increase of polyisoprene yield was highlighted by full conversion achieved in **2a** system with an obvious increase of molecular weight (*M*_n_ = 22.3 × 10^4^). Similarly, at large excess of MAO, **4a** and **5a** showed a same trend in the yields while multi-modal polyisoprene was generated.

#### 3.2.2. Polymerization of Isoprene with Co(II) Catalysts

Encouraged by the results in Fe(II) complexes catalyzed isoprene polymerization, further studies were carried out in Co(II) complexes using various common alkylaluminum reagents as cocatalysts. In iminopyridine Co(II)-catalyzed isoprene polymerization, MAO, Al(*i*-Bu)_3_, and AlEt_3_ showed an adverse effect on polymerization of isoprene (See Supplementary Materials, Table S4). However, AlEt_2_Cl was effective as cocatalyst. When the ratio of Al/Co was 500, no polymer was observed. Polymer appeared with the decrease of Al/Co ratio (See Supplementary Materials, Table S5). After optimization of Al/M ratio, Al/M = 10 was chosen as the optimal condition in Co(II)/AlEt_2_Cl binary system (See Table 2; NMR Spectra and GPC characterizations of the representative polyisoprene see Supplementary Materials, Figures S17, S18 and S30–S34). Co(II) complexes **1b** and **2b** containing electron-withdrawing aryl substituents afforded polymers with higher molecular weights at moderate yields (Table 2, entries 1 and 2) than those by the complex Co(II) containing electron-donating alkyl substituents (Table 2, entry 4: R = octyl). This is similar with the trend observed in the Fe(II)/MAO systems. At this stage, there is no correlation between the electronic effect of the complexes and the resultant polyisoprene, especially in the stereoselectivity.

#### 3.2.3. The Ternary System of Iminopyridine Fe(II) and Co(II)-Catalyzed Isoprene Polymerization

Raynaud et al. [27] reported a significant effect of introduction of a dealkylating reagent ([Ph_3_C][B(C_6_F_5_)_4_]) to the activated Fe(II) complexes using Al(*i*-Bu)_3_ and AlEt_3_ as cocatalyst. In our system, MAO/[Ph_3_C][B(C_6_F_5_)_4_] cocatalysts were proved to be effective to generate polyisoprene (See Table 3; Screening experiment with various cocatalysts see Supplementary Materials, Table S6; NMR Spectra and GPC characterizations of the representative polyisoprene see Supplementary Materials, Figures S19–S22 and S35–S43). The Fe(II) complexes having fluorine substituents showed high activities (Table 3, entries 1–3). Furthermore, the ratio between *trans*-1,4-/3,4- units was not affected by the substituents pattern. It is assumed that the cocatalysts play an important role in determining the stereoselectivity. In Co(II)-catalyzed system, the fluorinated aryl moiety is electronically more withdrawing than the alkyl moiety, which can reduce the electron density on the metal center, leading to better monomer coordination and faster chain propagation. This is supported by the fact that Co(II) complex **3b** bears the strongest electron-withdrawing substituent and displays the highest yield (71.8%). In addition, the molecular weight of polyisoprene obtained by fluorine-substituted complexes **1b**–**3b** is lower than the bulkier alkyl-substituted Co(II) complexes (**4b**) (R = octyl) (Table 3, 1400~1600 vs. 1600). Probably, the steric environment of the alkyl moiety retards chain transfer reaction more effectively than less hindered fluorine substituted aryl moiety. We have to mention that as for the resultant polyisoprene, there were miscellaneous peaks in aromatic areas from ^1^H NMR, which was speculated as by-product of toluene involved Friedel-Crafts reaction [45,46]. The change in stereoselectivity from binary system (*cis*-1,4-unit and 3,4-unit) to ternary system (*trans*-1,4-unit and 3,4-unit) is solely based on the cocatalysts. It is difficult to conclude the selectivity determining step because of the complexity of the cocatalyst and lack of reactive intermediates isolation. Furthermore, the isoprene coordination mode s-*cis* or s-*trans* (As is known for monoalkenes, the Cossee-Arlman mechanism is the main pathway for the formation of C–C bonds in the polymerization of alkenes. Such mechanism is true of conjugated diene polymerization by homogeneous single site catalysts involves two steps, namely coordination of the incoming monomer to the catalyst active site and subsequent monomer insertion into a metal-carbon bond. However, the polymerization mechanism for conjugated dienes presents several peculiar aspects, mainly related to the type of bond between the transition metal of the catalyst and the growing chain. This bond is of a σ type in monoalkene polymerizations, but is of the allylic type (*η*^3^) in conjugated diene polymerizations. Furthermore, the conjugated diene monomer can coordinate to the metal center assuming different conformations and hapticities: s-*cis*-*η*^4^ (*cis* conformation around the single bond and coordination of the two double bonds), s-*trans*-*η*^4^ (*trans* conformation around the single bond and coordination of the two double bonds), or s-*trans*-*η*^2^ (*trans* conformation around the single bond and coordination of only one double bond. More mechanistic details see Supplementary Materials Figure S45) [47], migratory insertion into an *η*^2^- or *η*^4^-coordinated isoprene, generation of *syn*- and *anti*-M-allyl intermediates, *syn*- to *anti*-rearrangements of the M-allyl complexes may all be relevant for selectivity [48,49,50].

## 4. Conclusions

In summary, we have investigated novel fluorinated aryl substituted iminopyridine Fe(II) and Co(II) complexes in isoprene polymerization. Higher polymer yield, high molecular weight and similar stereoselectivity (*cis*-1,4-/3,4-unit ≈ 1:1) were obtained when binary Fe/MAO system was employed. This is the same trend with Co/AlEt_2_Cl excepted the stereoselectivity (*cis*-1,4-/3,4-unit ≈ 7:3). In general, Fe(II) complexes exhibited higher activities than analogous Co(II) complexes. However, Co(II) complexes showed higher *cis*-1,4 selectivity than Fe(II) complexes under the same condition. When a dealkylating reagent [Ph_3_C][B(C_6_F_5_)_4_] was introduced in isoprene polymerization, low molecular weight (*M*_n_ = 1200~1700) and high *trans*-1,4 polyisoprene (>95%) were prepared using either Fe(II) or Co(II) catalysts and MAO cocatalyst. In such system, the differences of catalytic behaviors between Fe(II) and Co(II) systems were not obvious. These studies demonstrated that iminopyridine complexes containing fluorine substituents tended to enhance the catalytic activity and led to a relatively higher yield of polyisoprene. Fe(II) complex (**1a**) containing CF_3_ substituent represented a rare example of highly active Fe(II) catalyst and relatively low catalyst loading (1 μmol) in isoprene polymerization. In addition, it is observed that less amount of AlEt_2_Cl was required to initiate an effective isoprene polymerization in Co/AlEt_2_Cl binary system. The stereoselectivity of polyisoprene was strongly dependent on the cocatalysts type and metal center. Further studies aiming at exploring the controllable polymerization using iminopyridine complexes and mechanistic studies are now in progress in our laboratory.

## Figures and Tables

**Table 1:** Iminopyridine Fe(II)-catalyzed isoprene polymerization employed methylaluminoxane (MAO) as cocatalyst ^a^.

Entry	Complex	Yield (%)	Microstructure ^b^ (%)			*M*_n_^c^ (×10^−4^)	PDI ^c^	Activity ^d^
			*cis-*1,4	*trans-*1,4	3,4			
1	**1a**	>99.0	54	-	46	9.1	4.3	12.2
2	**2a**	85.3	54	-	46	9.7	3.5	7.2
3	**3a**	32.7	56	-	44	19.0	2.1	2.8
4	**4a**	10.9	-	90	10	62.7	2.1	0.9
						0.4	1.7	
5	**5a**	21.1	65	-	35	6.1	1.5	1.8

^a^ polymerization condition: Isoprene 2 mL, complex 8 µmol, Al/Fe = 500, 25 °C, toluene 5 mL, reaction time 2 h; ^b^ determined by ^1^H NMR and ^13^C NMR; ^c^ determined by gel permeation chromatograph (GPC); ^d^ 10^4^ g·(mol of Fe)^−1^·(h)^−1^.

**Table 2:** Iminopyridine Co(II)-catalyzed isoprene polymerization employed AlEt_2_Cl as cocatalyst ^a^.

Entry	Complex	Yield (%)	Microstructure ^b^ (%)		*M*_n_^c^ (×10^−4^)	PDI ^c^	Activity ^d^
			*cis-*1,4	3,4			
1	**1b**	60.2	73	27	14.0	1.8	2.6
2	**2b**	46.3	72	28	10.5	2.2	2.0
3	**3b**	29.1	71	29	8.0	3.0	1.2
4	**4b**	58.1	68	32	5.3	2.5	2.5
5	**5b**	21.3	73	27	13.9	2.1	0.9

^a^ general condition: Isoprene 2 mL, complex 8 µmol, 25 °C, toluene 5 mL, Al/Co = 10, reaction time 4 h; ^b^ determined by ^1^H NMR and ^13^C NMR; ^c^ determined by GPC; ^d^ 10^4^ g·(mol of Co)^−1^·(h)^−1^.

**Table 3:** Iminopyridine Fe(II) and Co(II)-catalyzed isoprene polymerization ^a^.

Entry	Complex	Yield (%)	Microstructure ^b^ (%)		*M*_n_^c^ (×10^−3^)	PDI ^c^	Activity ^d^
			*trans-*1,4	3,4			
1	**1a**	52.8	95	5	1.4	1.7	2.2
2	**2a**	76.3	98	2	1.5	2.1	3.2
3	**3a**	64.8	96	4	1.6	2.2	2.8
4	**4a**	30.2	98	2	1.5	1.8	1.3
5	**5a**	trace	-	-	-	-	-
6	**1b**	19.3	96	4	1.6	1.5	0.8
7	**2b**	39.7	98	2	1.4	1.7	1.7
8	**3b**	71.8	95	5	1.4	1.9	3.1
9	**4b**	19.6	98	2	1.6	1.5	0.8
10	**5b**	32.2	98	2	1.2	1.6	1.4

^a^ general condition: Isoprene 1 mL, Fe(II) or Co(II) complexes: 8 µmol, 25 °C, Al/Fe = Al/Co = 5, [Ph_3_C][B(C_6_F_5_)_4_]: 8 µmol, toluene 5 mL, reaction time 2 h; ^b^ determined by ^1^H NMR and ^13^C NMR; ^c^ determined by GPC; ^d^ 10^4^ g·(mol of Fe or Co)^−1^·(h)^−1^.

## Supplementary Materials

The following are available online at http://www.mdpi.com/2073-4360/10/9/934/s1, NMR spectra of the ligands **L3** (Figures S1–S3), HRMS-ESI of **L3** (Figure S4), TOF-MS-ES+ of Complexes (Figures S5–S10), NMR spectra of the representative polyisoprene (Figures S11–S24) and GPC curves of polyisoprene samples (Figures S25–S43), ion-paired cobalt complexes (Figure S44), Machanism of Formation of Polyisoprene (Figure S45), optimum condition screening polymerization (Tables S1–S6), crystal data of Fe(II) Complex **3a** (CCDC number: 1830611), Co(II) Complex **2b** (CCDC number: 1830608) and Co(II) Complex **3b** (CCDC number: 1830612) (Tables S7–S10).
