# Supplementary material for: Influences of Fluorine Substituents on Iminopyridine Fe(II)- and Co(II)-Catalyzed Isoprene Polymerization
Source: Polymers (Basel). 2018 Aug 22;10(9):934. doi: 10.3390/polym10090934 (PMC6403809; doi:10.3390/polym10090934)
Supplement: Supplementary file 1 [file polymers-10-00934-s001.pdf]

Supplementary Materials: Influences of Fluorine Substituents on Iminopyridine Fe(II)- and Co(II)-Catalyzed Isoprene Polymerization

Guangqian Zhu, Xianhui Zhang, Mengmeng Zhao, Liang Wang, Chuyang Jing, Peng Wang, Xiaowu Wang and Qinggang Wang

Table of Contents

1. Optimum Condition Screening Experiment.. 2
2. Characterization of Ligand **L3**... 5
3. TOF-MS-ES+ of Fe(II) and Co(II) complexes 6
4. NMR Spectra of the Representative Polyisoprene.............................. 8
5. GPC Characterization of Polyisoprene... 15
6. X-Ray Crystallography of Complexes... 25
7. Machanism of Formation of Polyisoprene... 33

1. Optimum Condition Screening Experiment

Table S1. Optimization in Fe(II) Complex **2a** Catalyzed Polymerization with Various Cocatalysts [a].

Entry	Cocatalyst	Al/Fe	Yield (%)	Microstructure [b] (%)	
				cis-1,4	3,4
1	MAO	500	85.2	54	46
2	Al(*i*-Bu)$_3$	500	18.5	-	
3	$AlEt_3$	500	3.8	-	
4	$AlEt_2Cl$	500	0.1	-	
5	$AlEtCl_2$	500	93.5	-	
6	SEAC	500	>99.0	-	

[a] general condition : Isoprene 2 mL, complex 8 μmol, 25 °C, toluene 5 mL, reaction time 2 h. [b] determined by ^{1}H NMR and ^{13}C NMR.

When **2a** was employed as catalyst, six cocatalysts were chosen in isoprene polymerization. From Table S1,we found that MAO led to an effective polymerization and stereoselectivity can be characterized by NMR (Table S1, entry 1); Al(*i*-Bu)$_3$ produced a complicated polymer which was unsolvable in most organic solvent such as CH_2Cl_2, THF, $CDCl_3$ (Table S1, entry 2); $AlEt_3$ and $AlEt_2Cl$ showed low activities in polymerization (Table S1, entries 3 and 4); $AlEtCl_2$ and EASC exhibited highest activity in isoprene polymerization, however, these polymers were unable to be characterized by NMR.

Table S2. Optimalized Isoprene Polymerization in **1a** [a].

Entry	Complex	Al/Fe	Yield (%)	Microstructure [b] (%)		M_n [c] (× 10^{-4})	PDI [c]	Activity [d]
				cis-1,4	3,4			
1	8	500	> 99.0	54	46	-	-	> 102
2	8	100	95.6	48	52	3.5	2.2	97.5
3	8	10	27.1	47	53	5.5	1.9	27.7
4[e]	8	10	34.6	46	54	10.9	3.9	8.8
5[f]	8	10	35.1	46	54	3.9	2.1	6.0
6	1	500	64.9	47	53	9.4<br>0.2	1.8<br>1.3	66.2
7	1	1000	69.0	47	53	18.4	2.86	70.3
8[e]	1	500	62.3	48	52	4.5	2.2	15.9
9[f]	1	500	64.9	47	53	21.1	2.1	11.0

[a] general condition : Isoprene 2 mL, complex 8 μmol, 25 °C, toluene 5 mL, reaction time 10 min. [b] determined by ^{1}H NMR and ^{13}C NMR; [c] determined by GPC; [d] 10^4 g·(mol of Fe)$^{-1}$·(h)$^{-1}$; [e] reaction time 40 min; [f] reaction time 1 h.

Table S3. Higher Al/Fe ratio effect on Optimalized Iminopyridine Fe(II)-catalyzed Isoprene Polymerizaton [a].

Entry	Complex	Yield (%)	Microstructure [b] (%)			M_n [c] (× 10^{-4})	PDI [c]	Activity [d]
			cis-1,4	*trans*-1,4	3,4			
1	2a	85.3	54	0	46	9.7	3.5	7.2
2	2a[e]	>99.0	54	0	46	22.3	2.8	8.5
3	3a	32.7	56	0	44	19.0	2.1	2.8
4	3a[e]	49.3	56	0	44	12.3	2.4	4.2
5	4a	10.9	0	90	10	62.7<br>0.4	2.1<br>1.7	0.9
6	4a[e]	42.0	0	80	20	18.7<br>0.2	2.3<br>2.5	4.5
7	5a	21.1	65	0	35	6.1	1.5	1.8
8	5a[e]	28.8	65	0	35	53.0<br>3.3<br>1.1	1.3<br>1.8<br>1.5	2.5

[a] polymerization condition: Isoprene 2 mL, complex 8 μmol, Al/Fe = 500, 25 °C, toluene 5 mL, reaction time 2 h; [b] determined by ^1H NMR and ^{13}C NMR; [c] determined by GPC; [d] 10^4 g·(mol of Fe)$^{-1}$·(h)$^{-1}$; [e] Al/Fe = 1000.

Table S4. The Influences on isoprene polymerization using iminopyridine Co(II) **2b** with different cocatalysts [a].

Cocatalyst	Al/M	Yield (%)	Microstructure [b] (%)		M_n [c] (× 10^{-4})
			cis-1,4	3,4	
$AlEt_2Cl$	500	0	-		-
$AlEt_2Cl$	100	trace	-		-
$AlEt_2Cl$	25	24.3	71	29	4.0
$AlEt_2Cl$	10	46.3	72	28	10.5
Al(*i*-Bu)$_3$	500	27.1	unsolvable		-
Al(*i*-Bu)$_3$	100	trace	-		-
Al(*i*-Bu)$_3$	25	0	-		-
Al(*i*-Bu)$_3$	10	0	-		-
$AlEt_3$	500	0	-		-
$AlEt_3$	100	0	-		-
$AlEt_3$	25	0	-		-
$AlEt_3$	10	0	-		-
MAO	500	0	-		-
MAO	100	0	-		-
MAO	25	0			-
MAO	10	0	-		-

[a] general condition : Isoprene 2 mL, complex 8 μmol, 25 °C, toluene 5 mL, reaction time 2 h. [b] determined by ^1H NMR and ^{13}C NMR; [c] determined by GPC.

When employed $AlEt_2Cl$ as cocatalyst, low ratio of Al/Co catalyzed polymerization generated polyisoprene with relatively high *cis*-1,4 units and high molecular weight. Meanwhile, the yield and the molecular weight of generated polyisoprene had a tendency to increase with a decrease amount of $AlEt_2Cl$ (Table S4). In order to prove the tendency, we developed the controlled experiments showed as Table S5.

Table S5. Low $AlEt_2Cl$/Co ratio effects on isoprene polymerization employing **1b**–**5b** [a].

Complex	Al/Co	Yield (%)	Microstructure [b] (%)		M_n [c] (× 10^{-4})	PDI [c]
			cis-1,4	3,4		
1b	25	46.0	73	27	8.4	1.8
1b	10	60.2	73	27	14.0	1.8
2b	25	24.3	71	29	4.0	2.1
2b	10	46.3	72	28	10.5	2.2
3b	25	20.6	71	29	4.5	2.3
3b	10	29.1	71	29	8.0	3.0
4b	25	52.2	70	30	4.3	2.5
4b	10	57.8	68	32	5.3	2.5
5b	25	19.1	72	28	6.8	2.1
5b	10	21.3	73	27	10.5	2.2

[a] general condition : Isoprene 2 mL, complex 8 μmol, 25 °C, toluene 5 mL, reaction time 2 h. [b] determined by ^{1}H NMR and ^{13}C NMR; [c] determined by GPC.

When the ratio of Al/Co reduced from 25 to 10, all generated polyisoprene showed an increase in both yield and molecular weight. These indicated that the decrease of $AlEt_2Cl$ constricted chain transfer reaction, which led to an increase of molecular weight.

Table S6. Optimization in Fe(II) complex **1a** catalyzed polymerization with various cocatalysts [a].

Cocatalyst	Al/M	B/M	Yield (%)	Microstructure [b] (%)		M_n [c]
				Trans-1,4	3,4	(× 10^{-3})
MAO	5	1	52.8	95	5	1.4
Al(*i*-Bu)$_3$	5	1	0	-	-	-
$AlEt_3$	5	1	0	-	-	-
$AlEt_2Cl$	5	1	0	-	-	-

[a] general condition : Isoprene 1 mL, Fe(II) or Co(II) complexes: 8 μmol, 25 °C, Al/Fe = Al/Co = 5, $[Ph_3C][B(C_6F_5)_4]$: 8 μmol, toluene 5 mL, reaction time 2 h; [b] determined by ^{1}H NMR and ^{13}C NMR; [c] determined by GPC.

For ternary system, four cocatalyst were studied in the polymerization with the introduction of $[Ph_3C][B(C_6F_5)_4]$. After screening, MAO was affirmed as an effective cocatalyst.

2. Characterization of Ligand L3

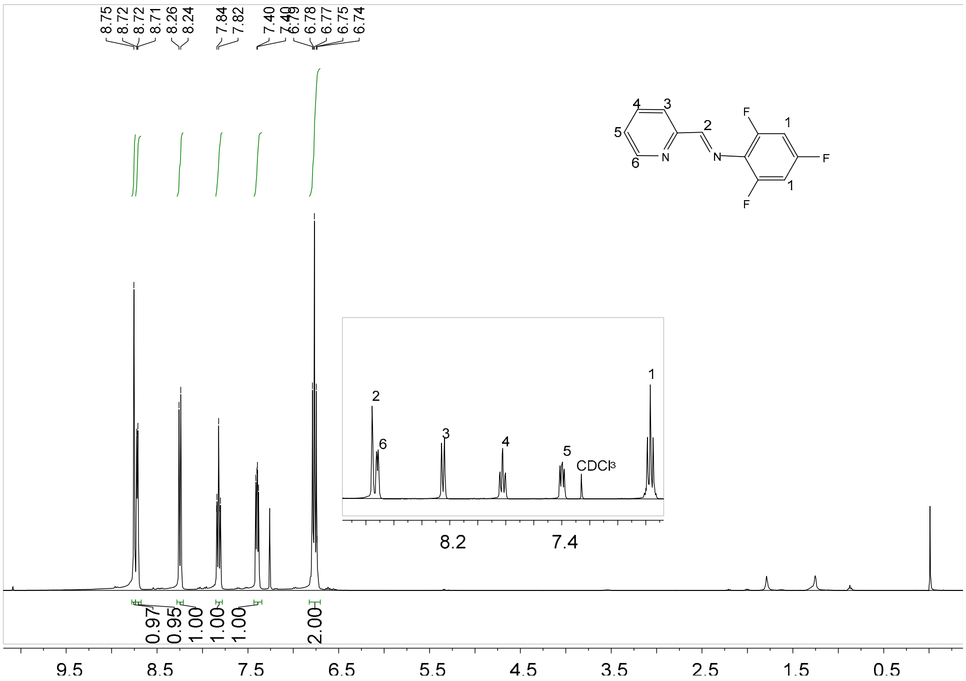

Figure S1. ^{1}H NMR spectrum (400 MHz, $CDCl_3$, 298 K) of the ligand **L3**.

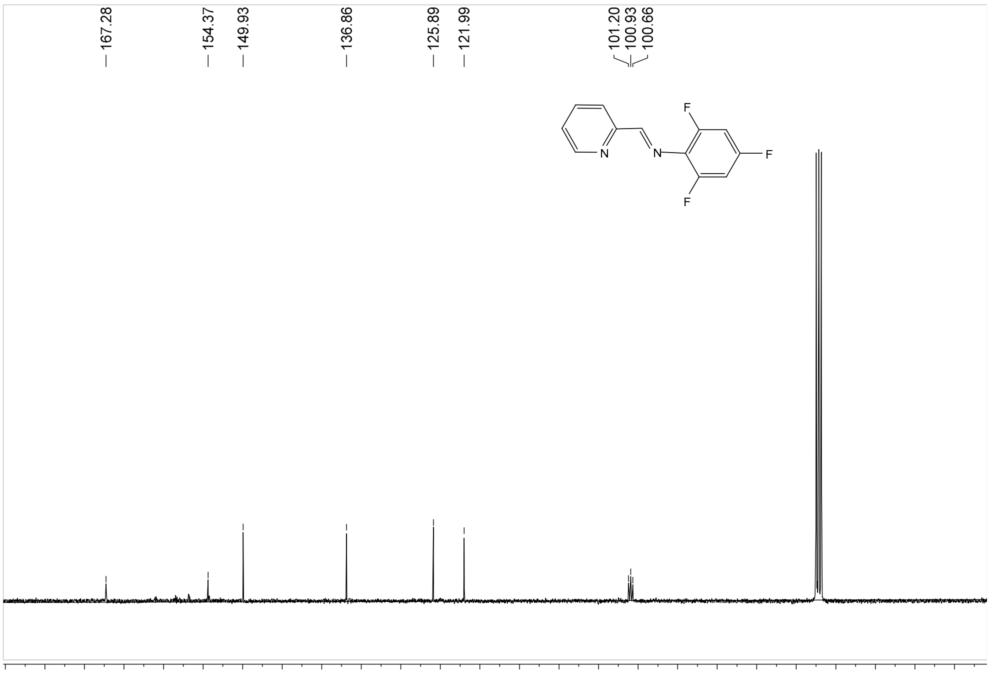

Figure S2. $^{13}C\{^{1}H\}$ NMR spectrum (100 MHz, $CDCl_3$, 298 K) of ligand **L3**.

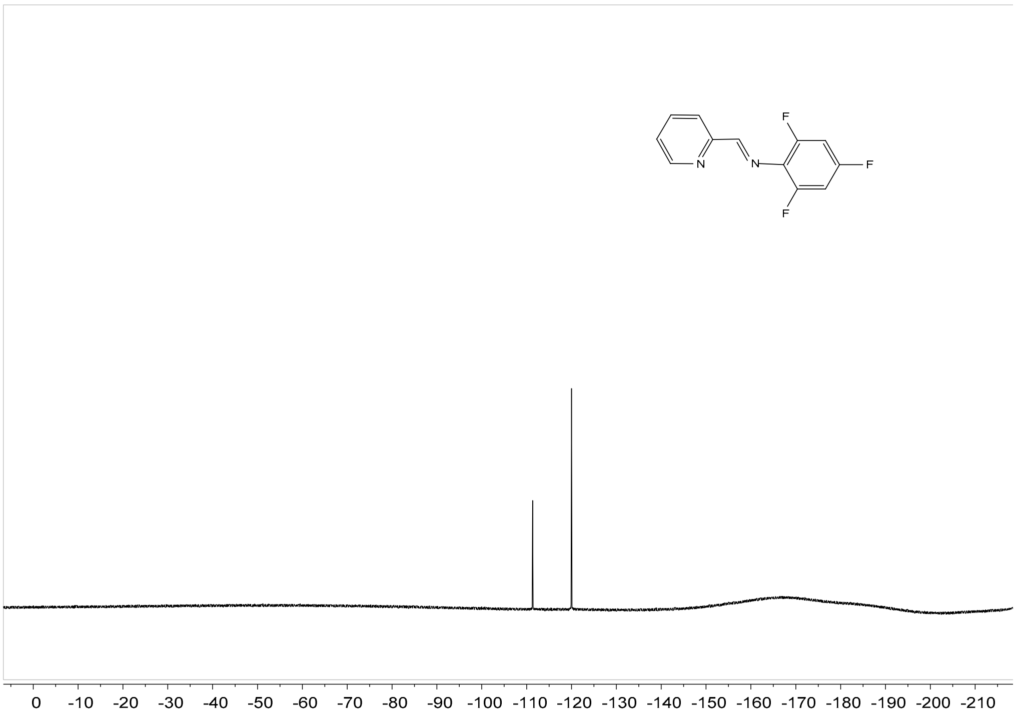

Figure S3. ^{19}F NMR spectrum (376 MHz, $CDCl_3$, 298 K) of ligand **L3** .

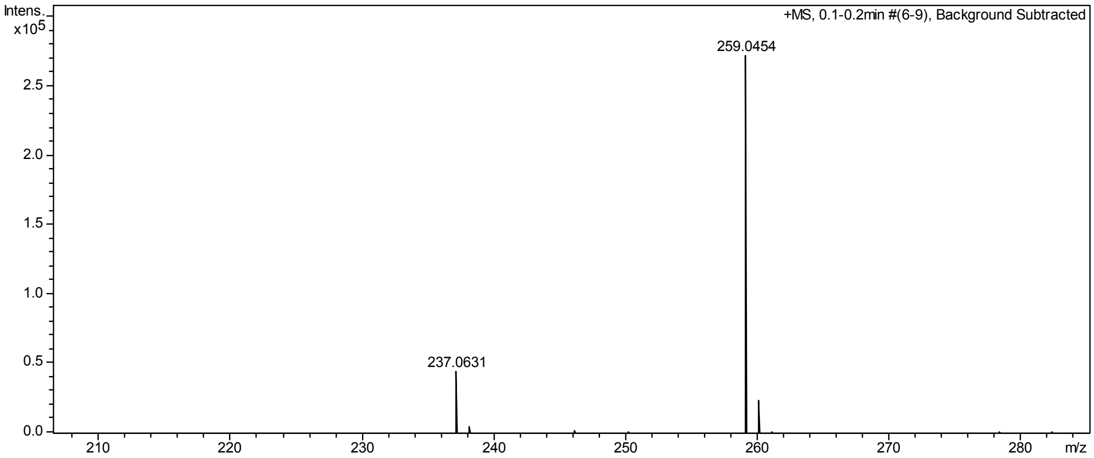

Figure S4. HRMS-ESI of ligand **L3** in CH_3CN.

3. TOF-MS-ES+ of Fe(II) and Co(II) Complexes

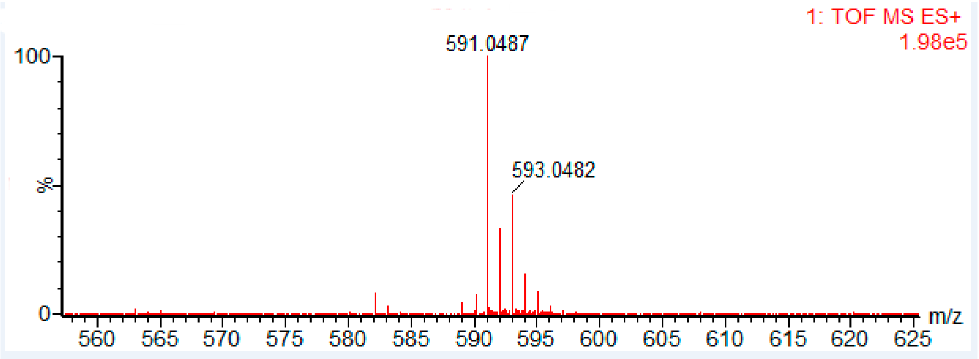

Figure S5. TOF-MS-ES+ of complex **1a.**

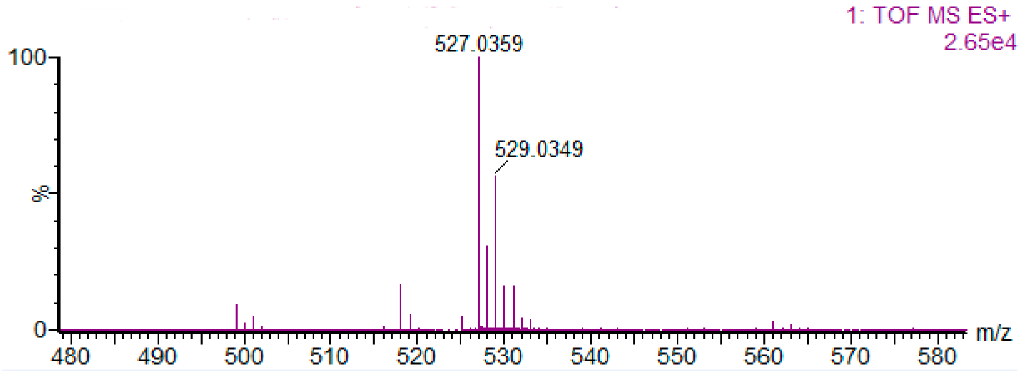

Figure S6. TOF-MS-ES+ of complex **2a.**

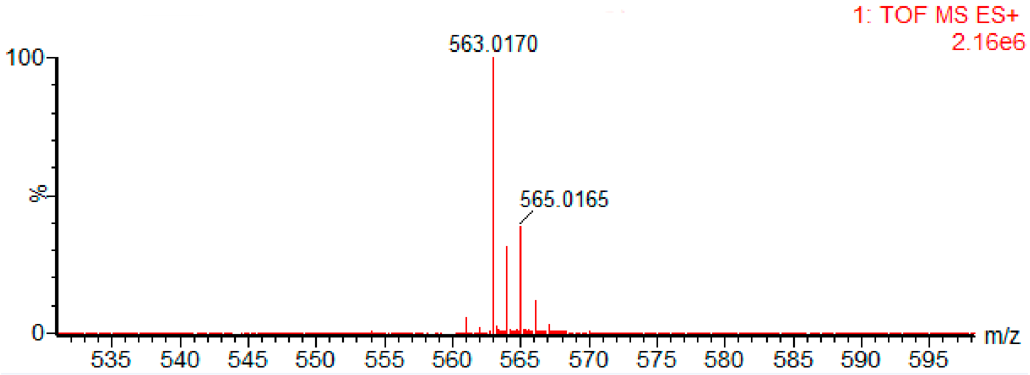

Figure S7. TOF-MS-ES+ of complex **3a.**

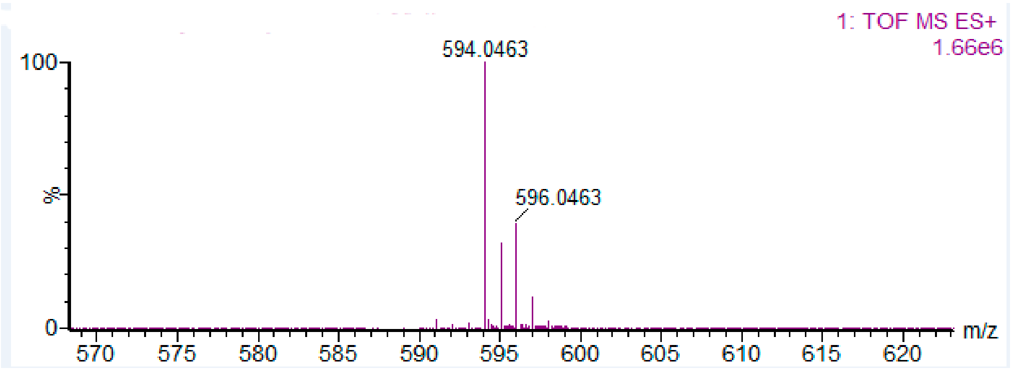

Figure S8. TOF-MS-ES+ of complex **1b.**

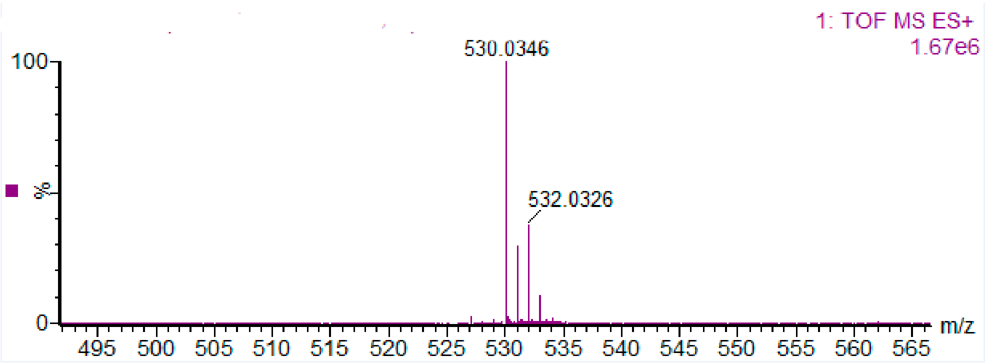

Figure S9. TOF-MS-ES+ of complex **2b.**

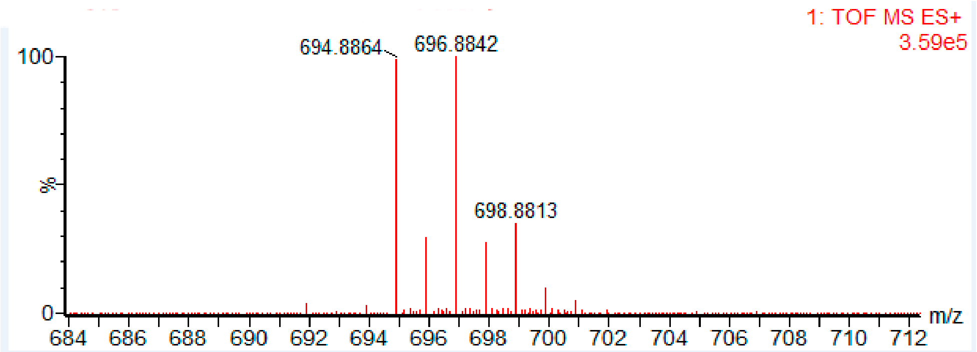

Figure S10. TOF-MS-ES+ of complex **3b.**

4. NMR Spectra of the Representative Polyisoprene

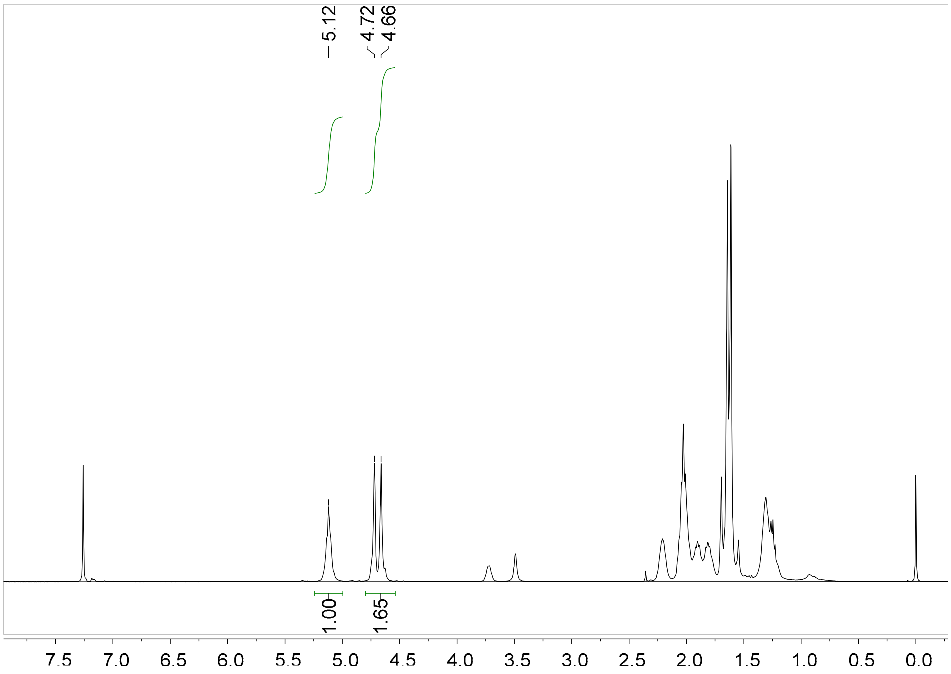

Figure S11. ^{1}H NMR spectrum (400 MHz, $CDCl_3$, 298 K) of polyisoprene (Table 1, entry 2).

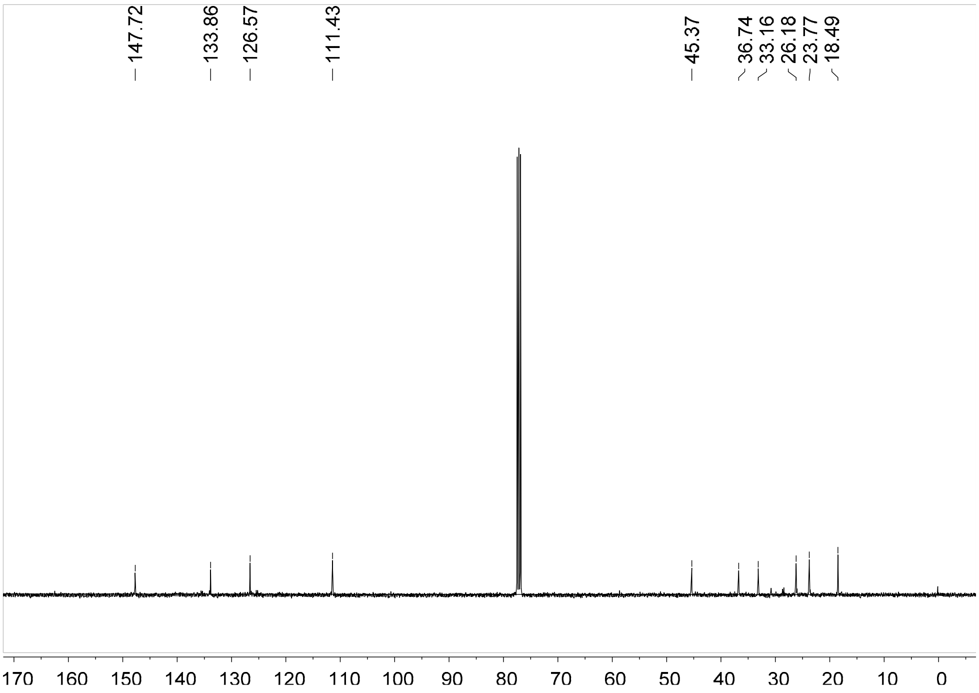

Figure S12. $^{13}C\{^1H\}$ NMR spectrum (100 MHz, $CDCl_3$, 298 K) of polyisoprene (Table 1, entry 2).

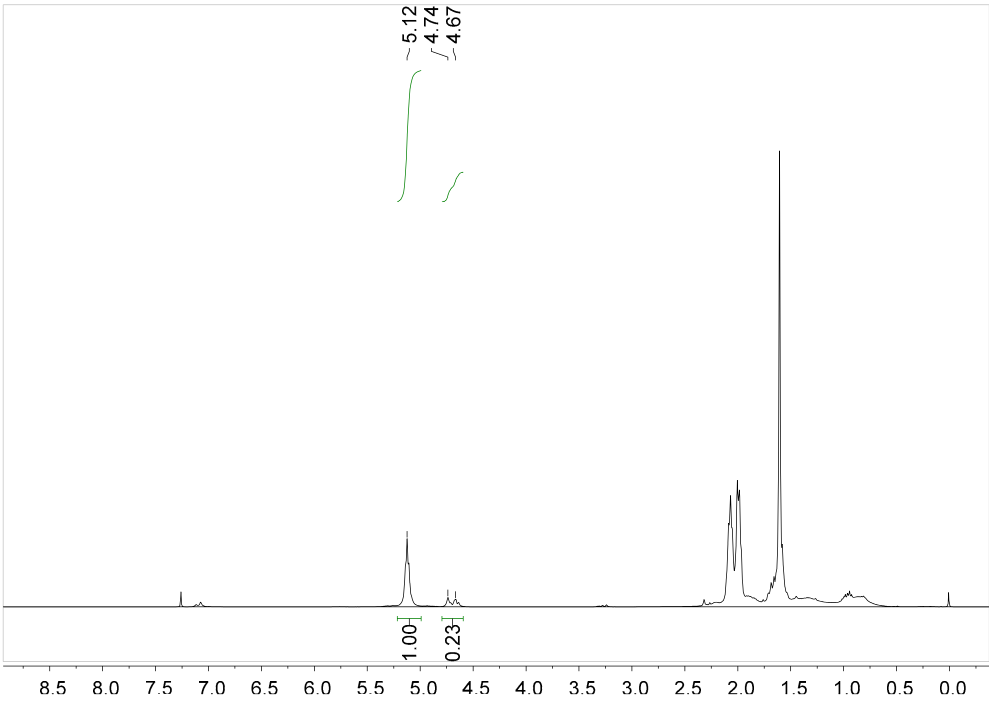

Figure S13. ^1H NMR spectrum (400 MHz, $CDCl_3$, 298 K) of polyisoprene (Table 1, entry 4).

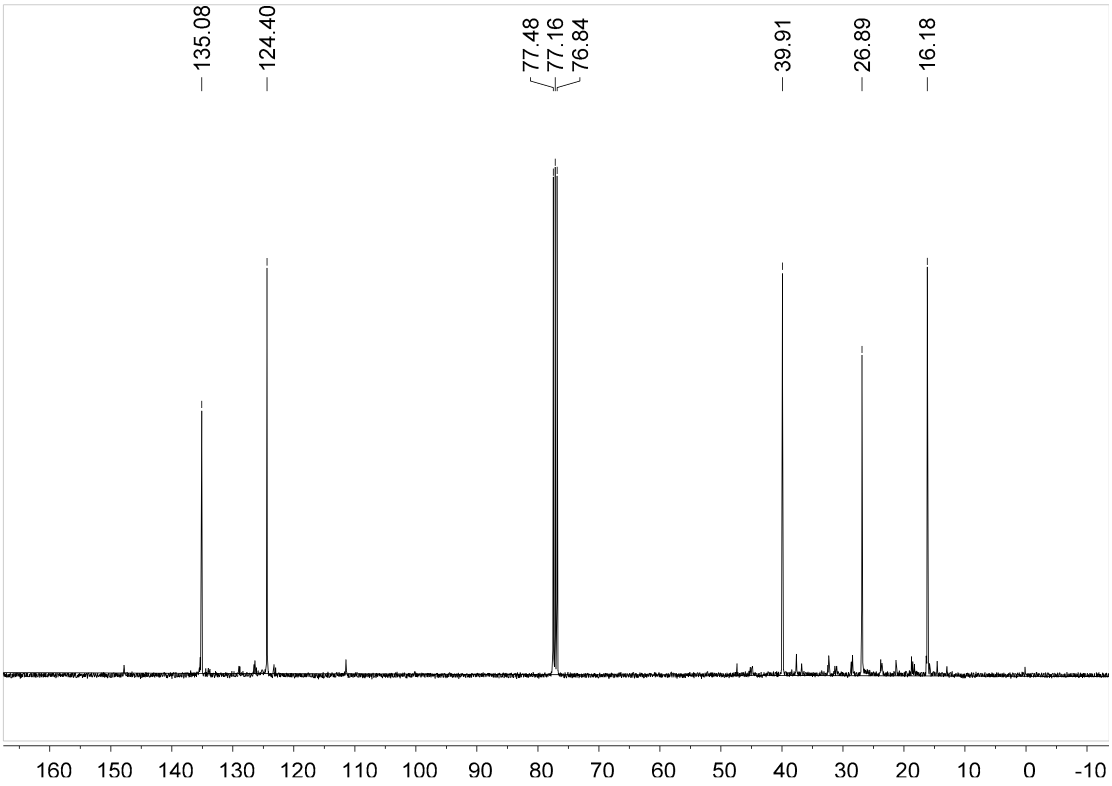

Figure S14. $^{13}C\{^{1}H\}$ NMR spectrum (100 MHz, $CDCl_3$, 298 K) of polyisoprene (Table 1, entry 4).

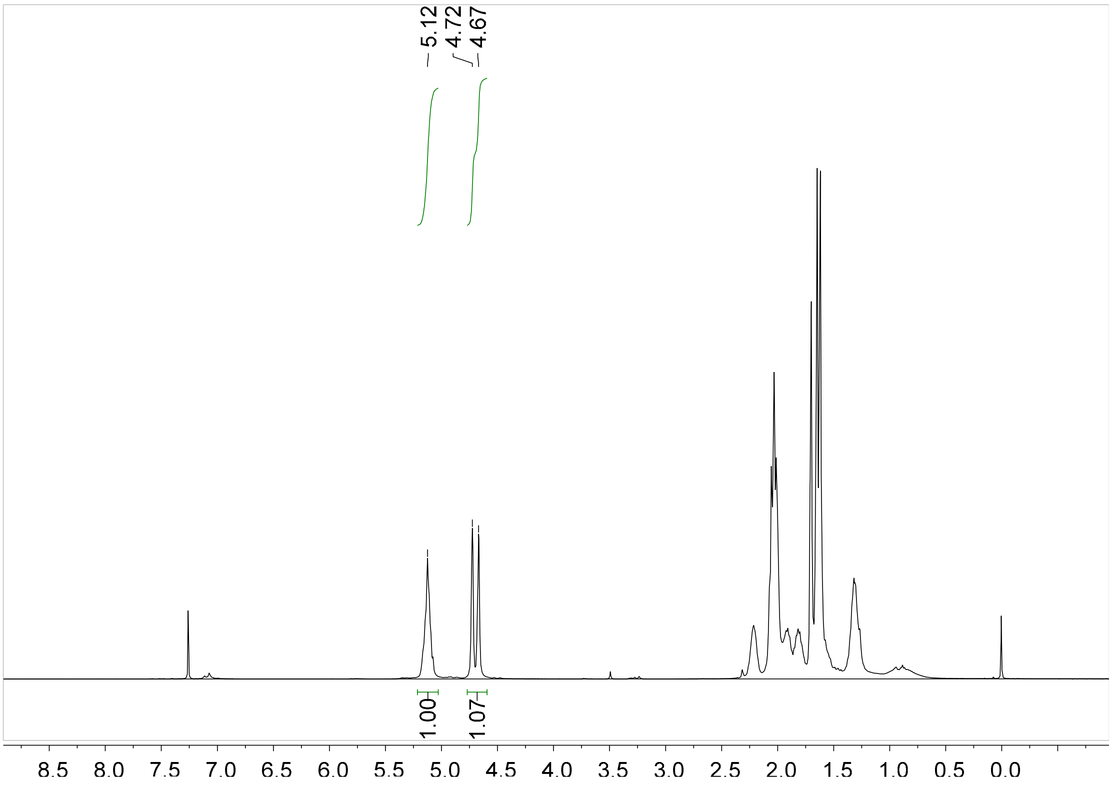

Figure S15. ^{1}H NMR spectrum (400 MHz, $CDCl_3$, 298 K) of polyisoprene (Table 1, entry 5).

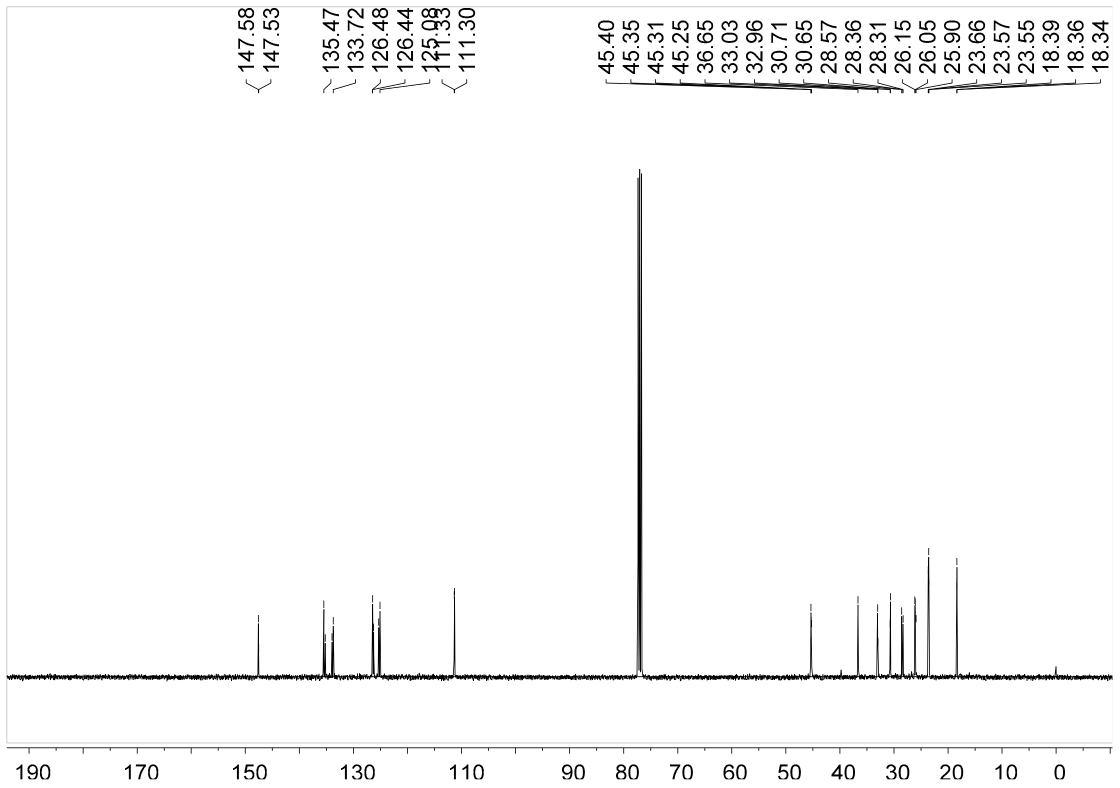

Figure S16. $^{13}C\{^1H\}$ NMR spectrum (100 MHz, $CDCl_3$, 298 K) of polyisoprene (Table 1,entry 5).

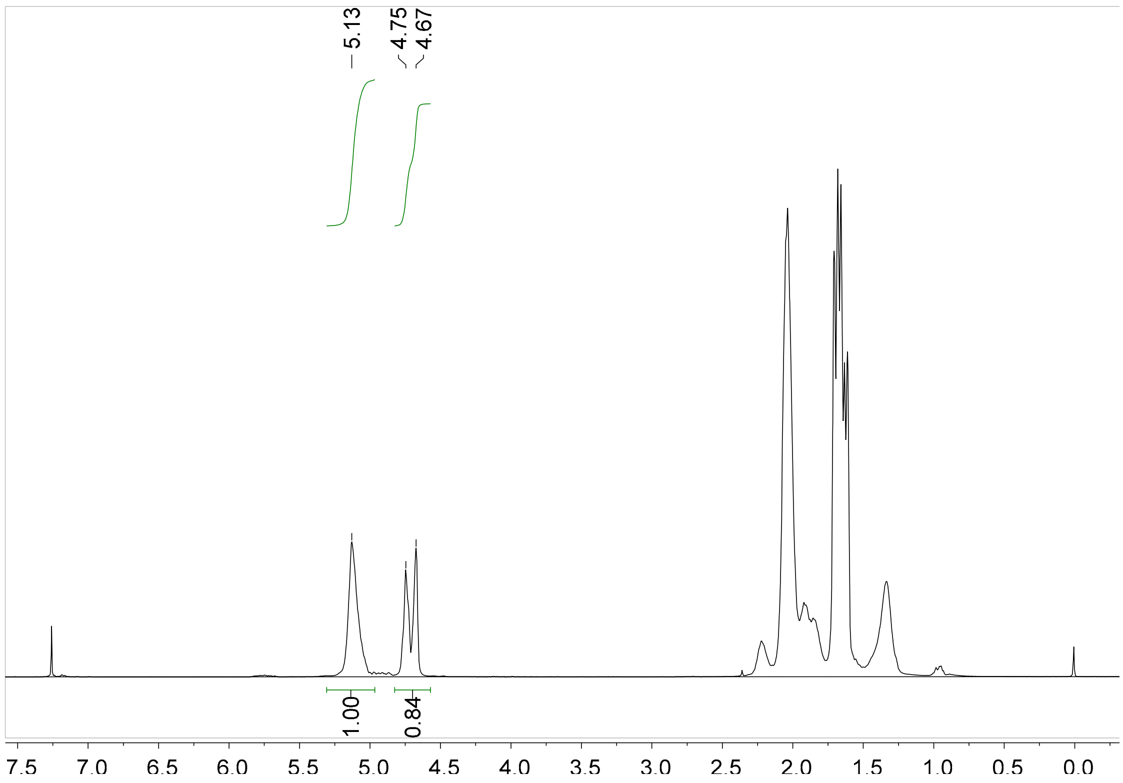

Figure S17. ^1H NMR spectrum (400 MHz, $CDCl_3$, 298 K) of polyisoprene (Table 2, entry 3).

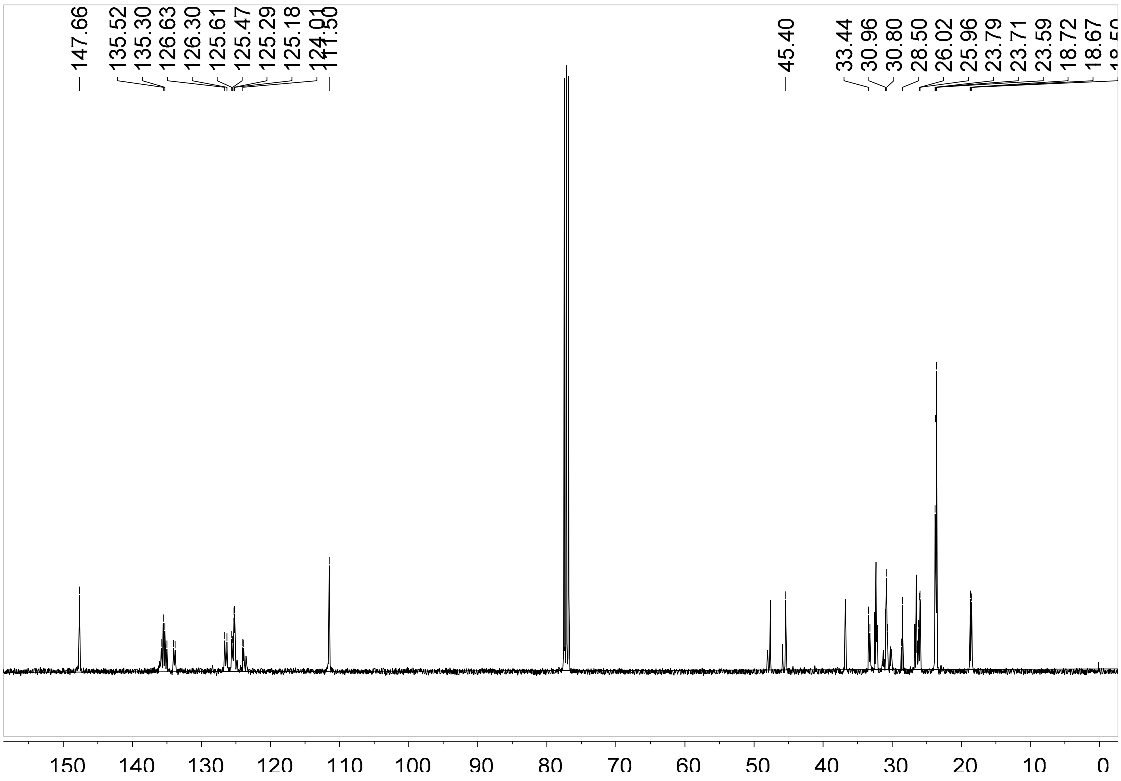

Figure S18. $^{13}C\{^{1}H\}$ NMR spectrum (100 MHz, $CDCl_3$, 298 K) of polyisoprene (Table 2, entry 3).

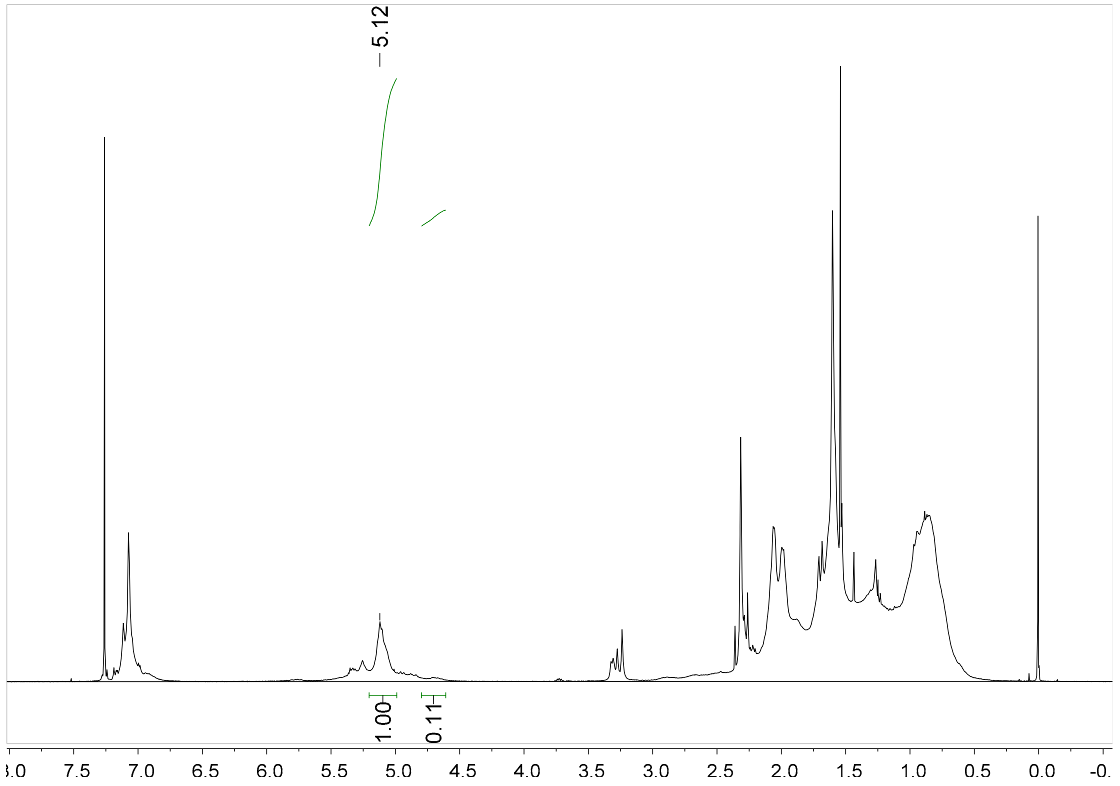

Figure S19. ^{1}H NMR spectrum (400 MHz, $CDCl_3$, 298 K) of polyisoprene (Table 3, entry 1).

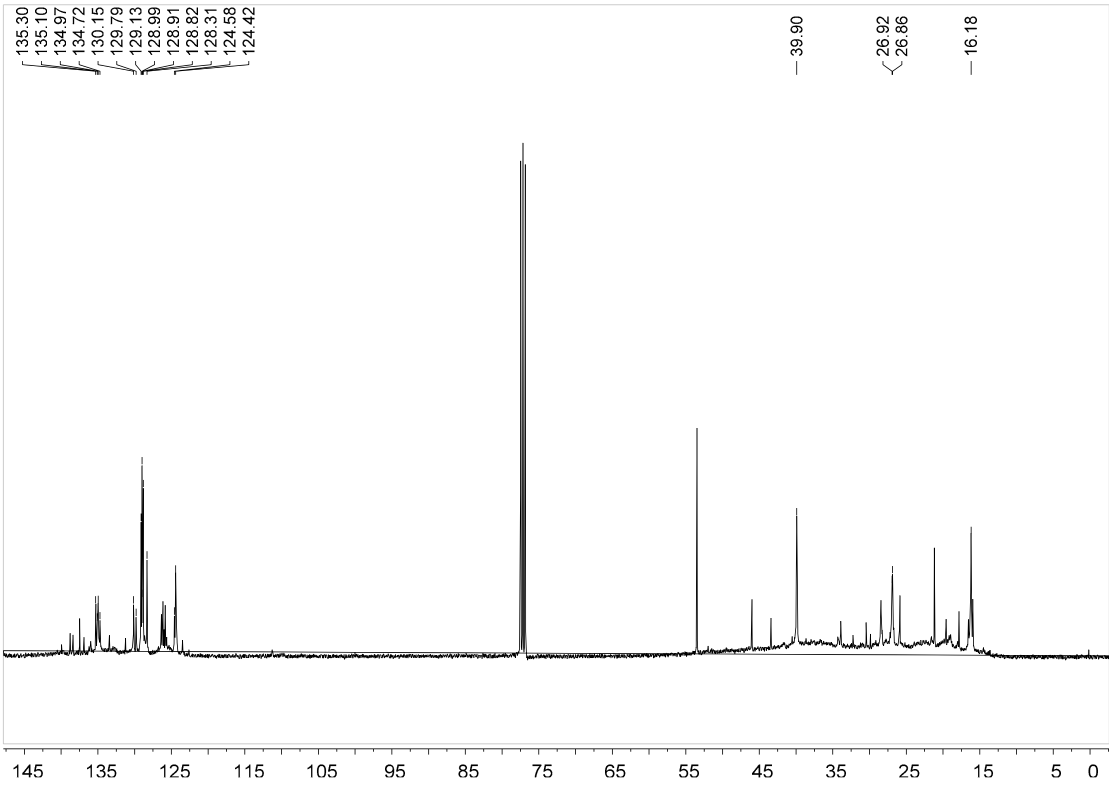

Figure S20. $^{13}C\{^1H\}$ NMR spectrum (100 MHz, $CDCl_3$, 298 K) of polyisoprene (Table 3, entry 1).

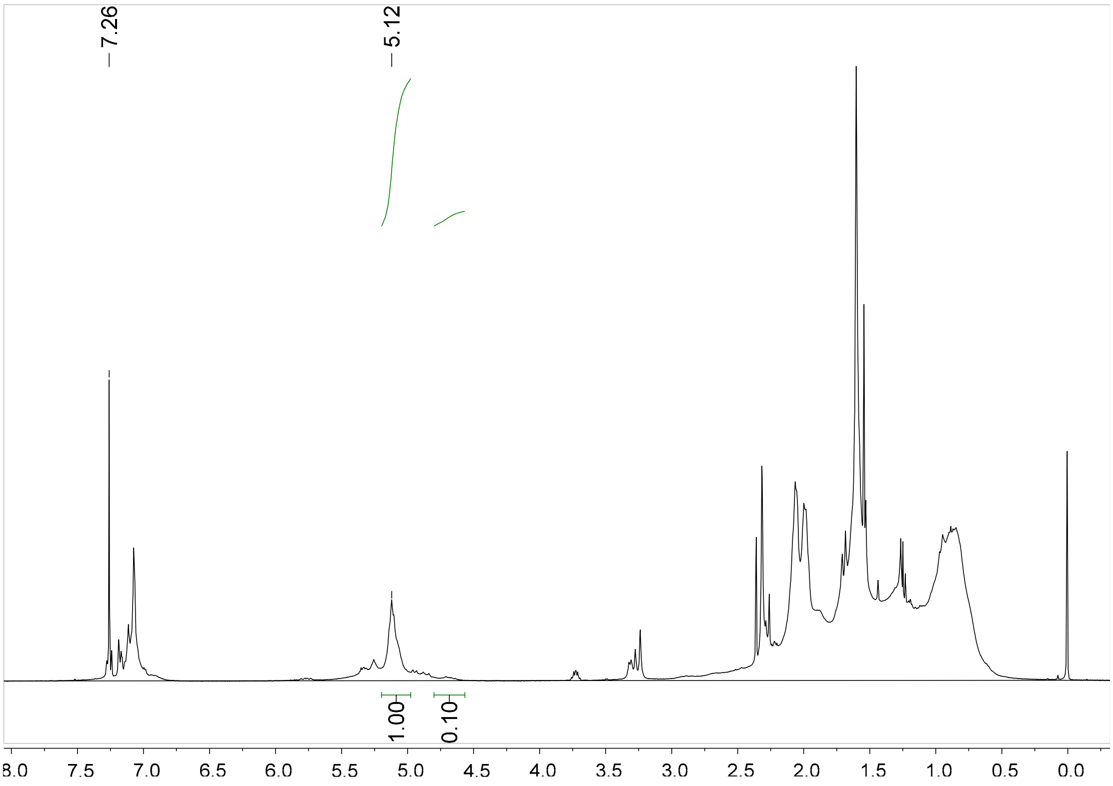

Figure S21. ^1H NMR spectrum (400 MHz, $CDCl_3$, 298 K) of polyisoprene (Table 3, entry 6).

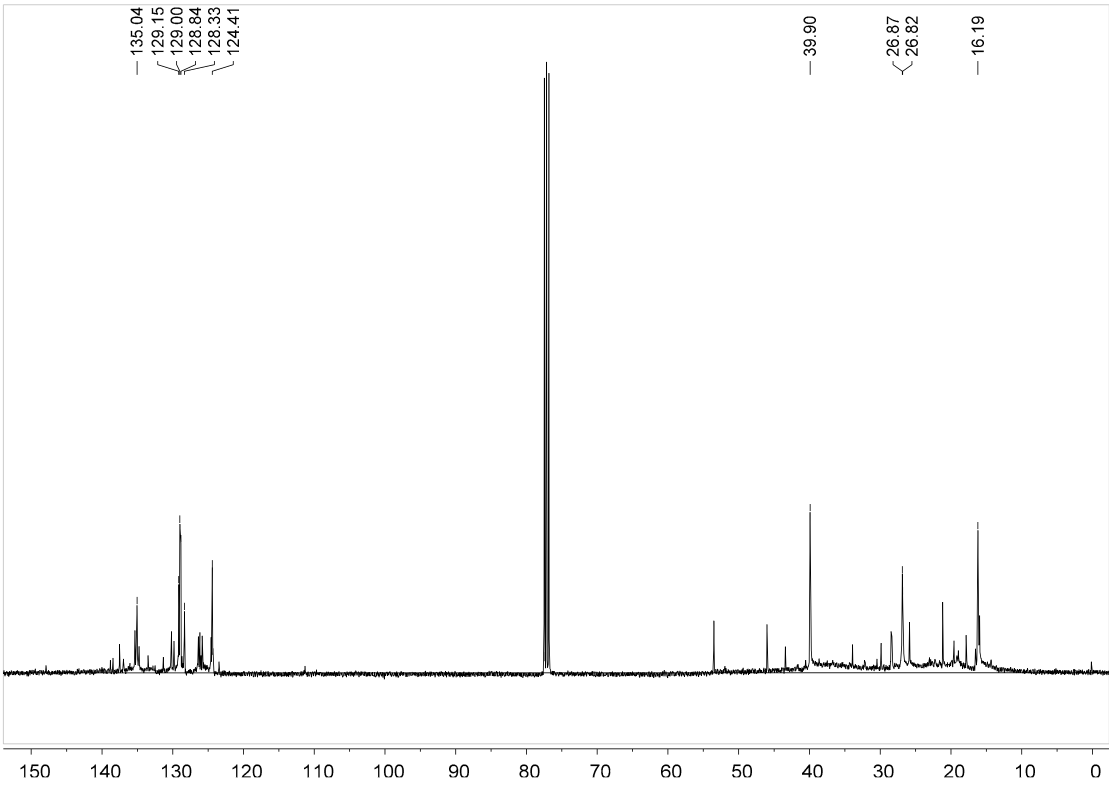

Figure S22. $^{13}C\{^{1}H\}$ NMR spectrum (100 MHz, $CDCl_3$, 298 K) of polyisoprene (Table 3, entry 6).

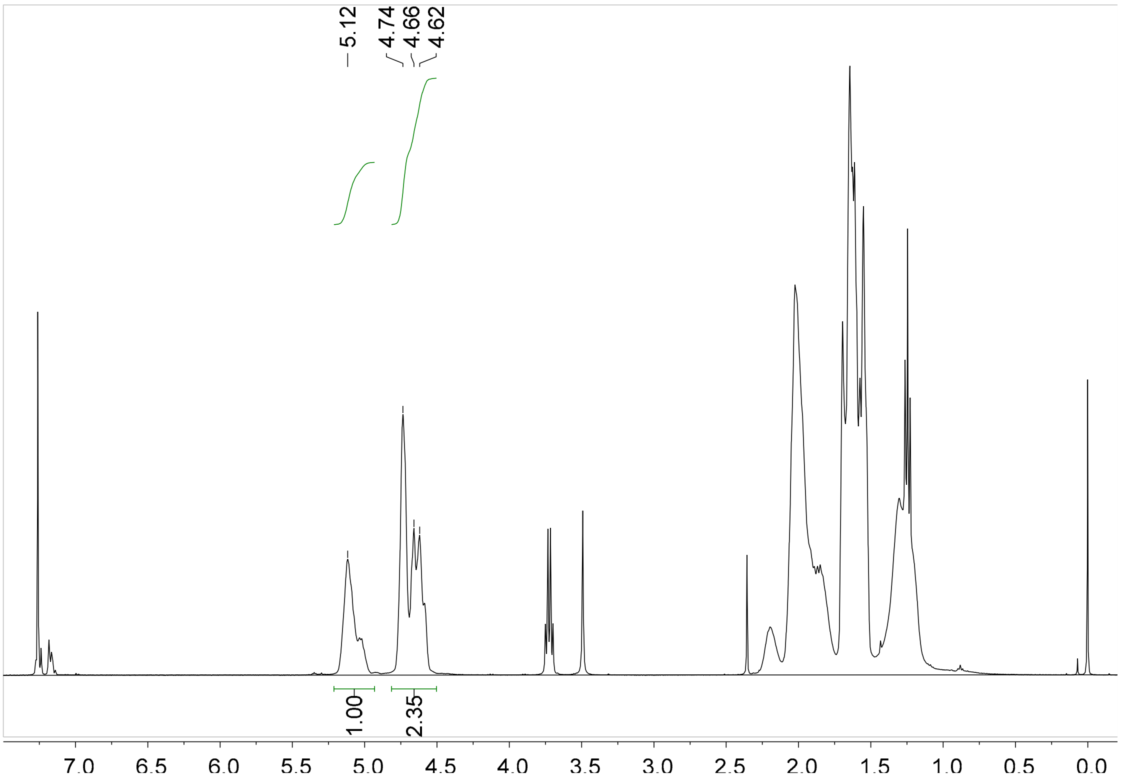

Figure S23. ^{1}H NMR spectrum (400 MHz, $CDCl_3$, 298 K) of polyisoprene (Table S2, entry 5).

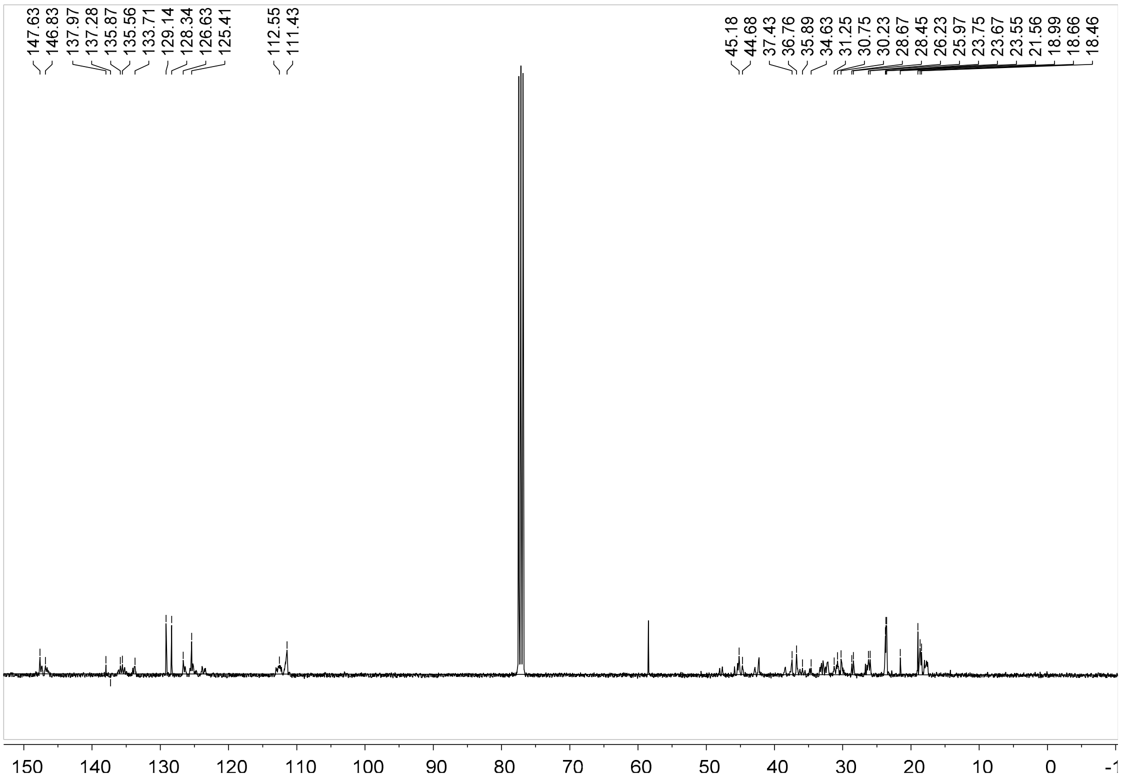

Figure S24. $^{13}C\{^1H\}$ NMR spectrum (100 MHz, $CDCl_3$, 298 K) of polyisoprene (Table S2, entry 5).

5. GPC Characterization of Polyisoprene

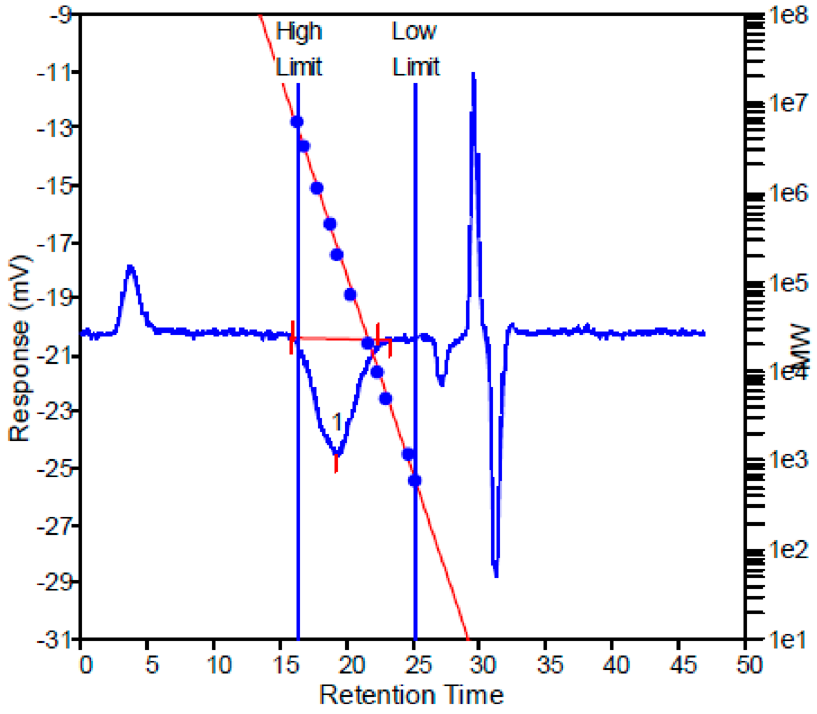

MW Averages

Peak No	Mp	Mn	Mw	Mz	Mz+1	Mv	PD
1	184887	91243	396756	1118520	1922584	332289	4.34834

Figure S25. the GPC of Fe(II) complex **1a** catalyzed polyisoprene in binary catalytic system (Table 1, entry 1).

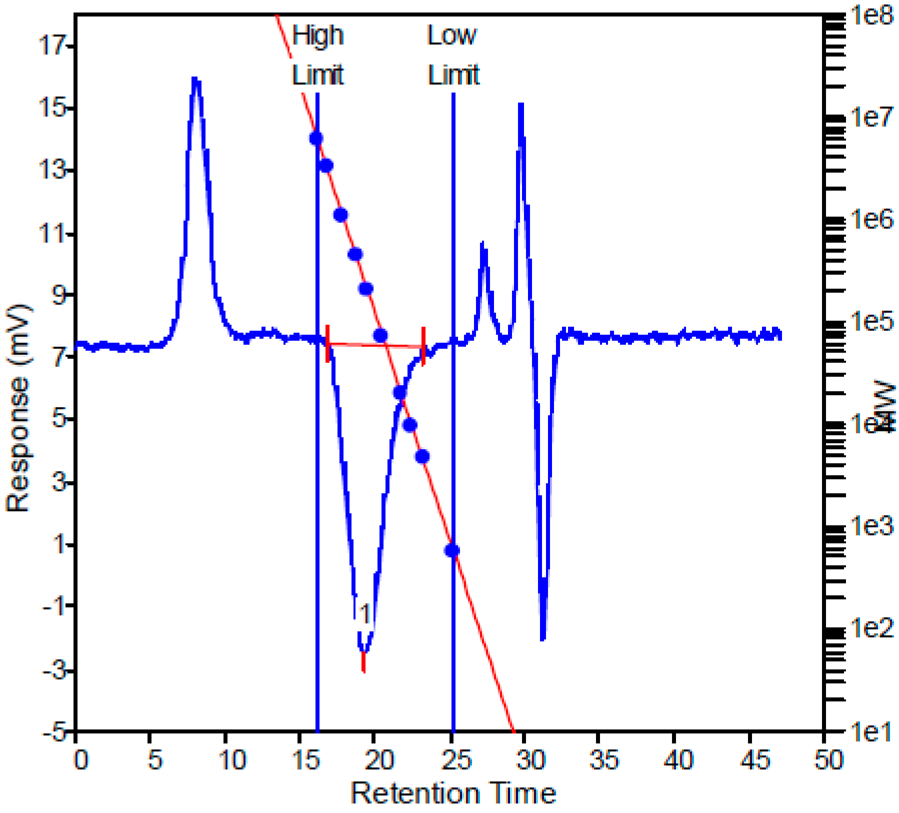

MW Averages

Peak No	Mp	Mn	Mw	Mz	Mz+1	Mv	PD
1	257280	97457	348153	750995	1174217	296950	3.57238

Figure S26. the GPC of Fe(II) complex **2a** catalyzed polyisoprene in binary catalytic system (Table 1, entry 2).

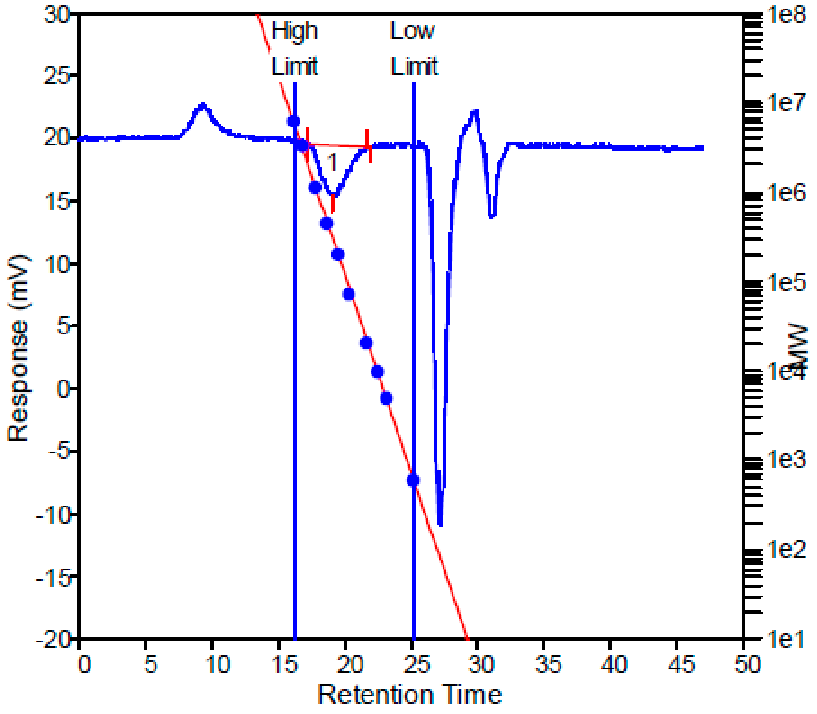

MW Averages

Peak No	Mp	Mn	Mw	Mz	Mz+1	Mv	PD
1	320812	189516	395674	667333	928773	356929	2.08781

Figure S27. the GPC of Fe(II) complex **3a** catalyzed polyisoprene in binary catalytic system (Table 1, entry 3).

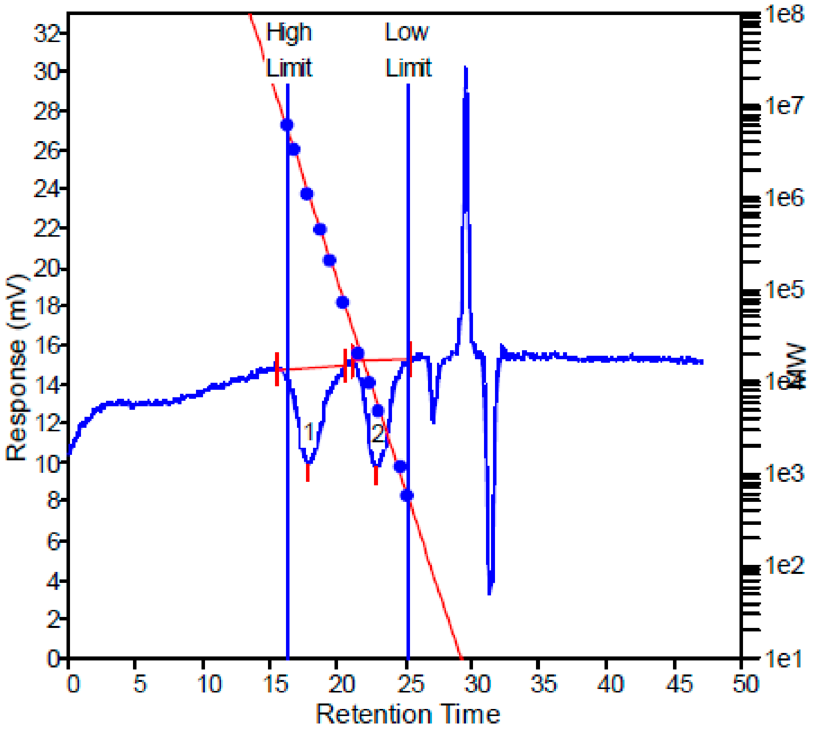

MW Averages

Peak No	Mp	Mn	Mw	Mz	Mz+1	Mv	PD
1	1155670	626886	1330046	2316112	3404329	1196115	2.12167
2	6302	4149	7327	11360	15606	6749	1.76597

Figure S28. the GPC of Fe(II) complex **4a** catalyzed polyisoprene in binary catalytic system (Table 1, entry 4).

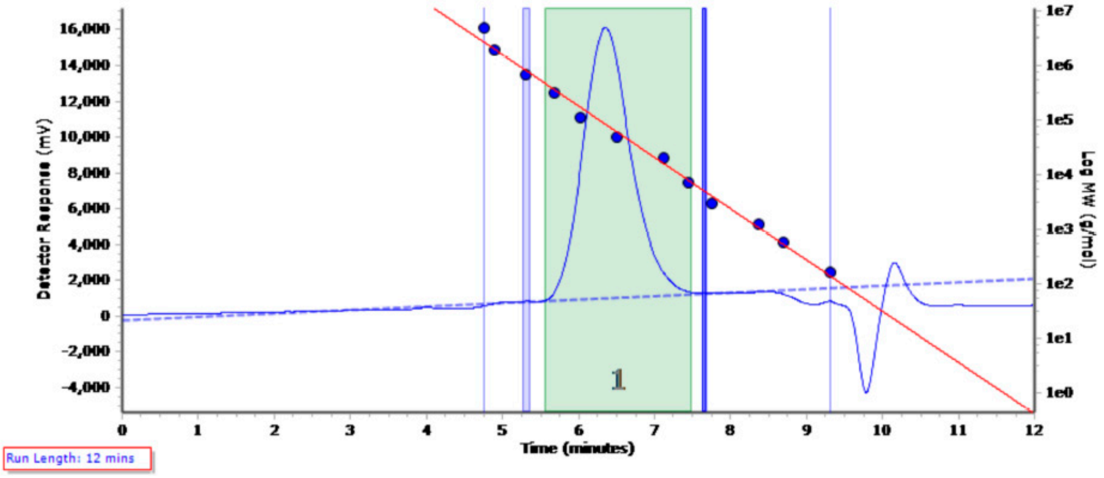

Molecular Weight Averages

Peak	Mp	Mn	Mw	Mz	Mz+1	Mv	PD
Peak 1	85012	60595	92797	130006	169657	124393	1.531

Figure S29. the GPC of Fe(II) complex **5a** catalyzed polyisoprene in binary catalytic system (Table 1, entry 5).

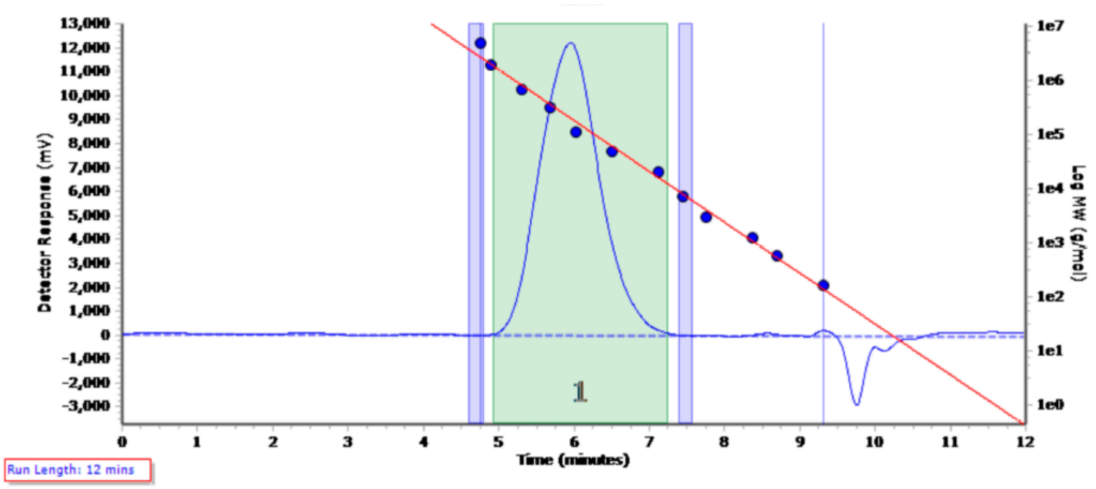

Molecular Weight Averages

Peak	Mp	Mn	Mw	Mz	Mz+1	Mv	PD
Peak 1	202317	139639	263102	430515	619443	404523	1.884

Figure S30. the GPC of Co(II) complex **1b** catalyzed polyisoprene in binary catalytic system (Table 2, entry 1).

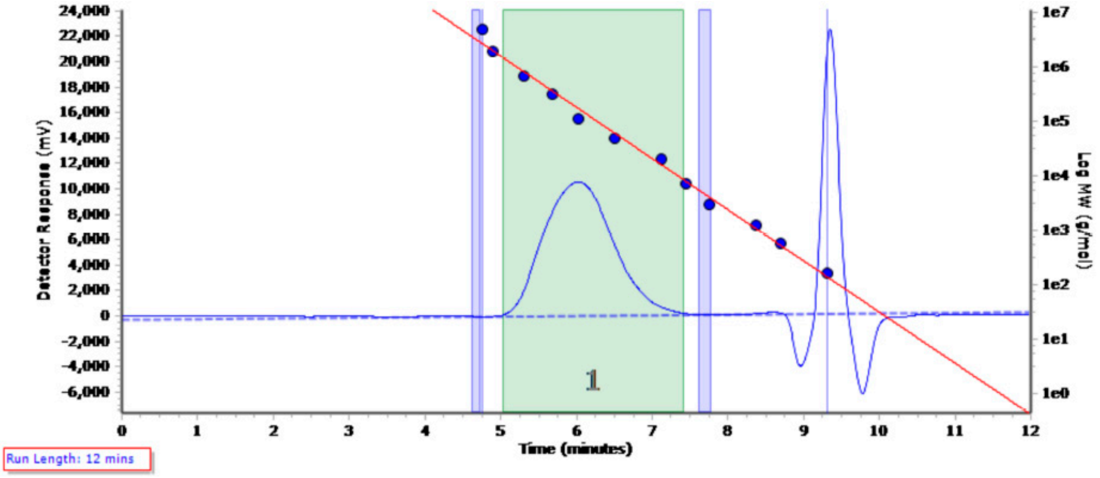

Molecular Weight Averages

Peak	Mp	Mn	Mw	Mz	Mz+1	Mv	PD
Peak 1	171961	104794	230472	402779	579374	377374	2.199

Figure S31. the GPC of Co(II) complex **2b** catalyzed polyisoprene in binary catalytic system (Table 2, entry 2).

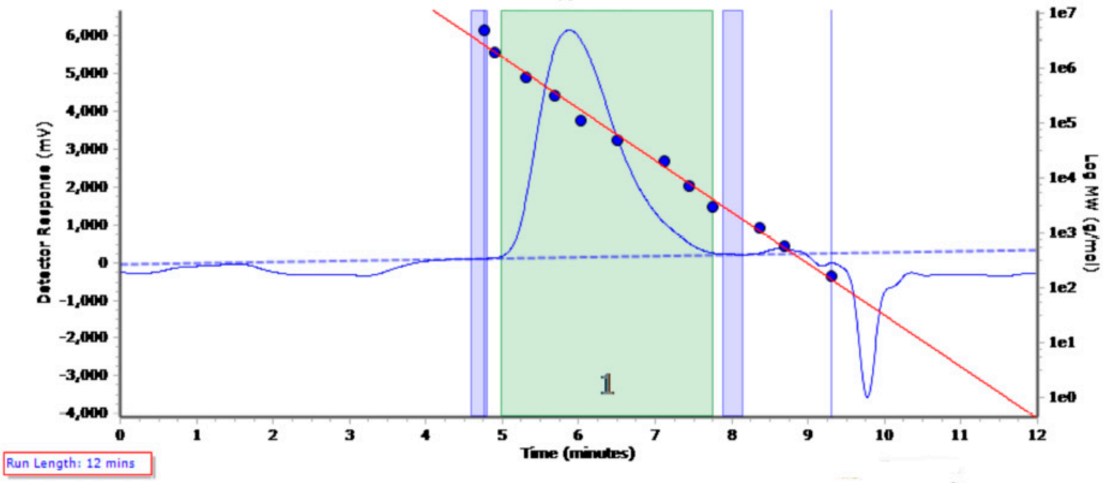

Molecular Weight Averages

Peak	Mp	Mn	Mw	Mz	Mz+1	Mv	PD
Peak 1	242371	79598	238960	434148	614395	407698	3.002

Figure S32. the GPC of Co(II) complex **3b** catalyzed polyisoprene in binary catalytic system (Table 2, entry 3).

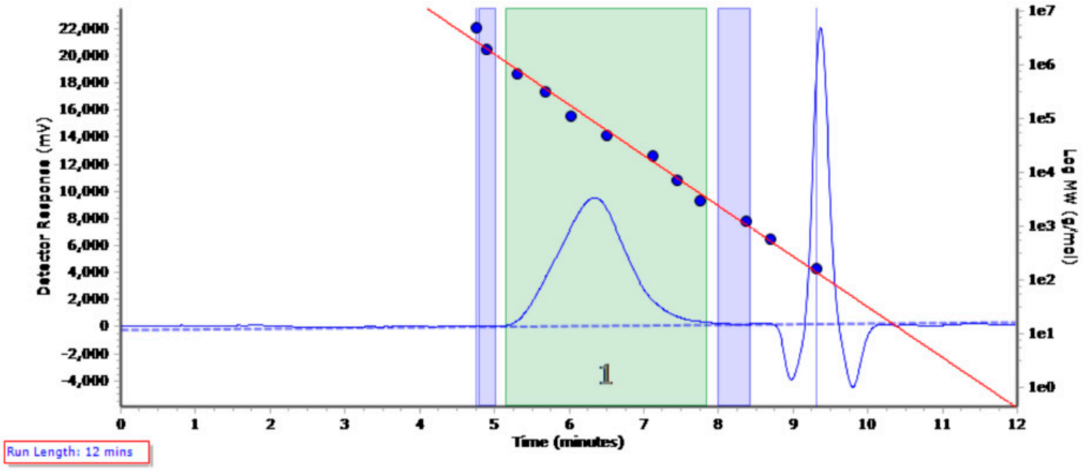

Molecular Weight Averages

Peak	Mp	Mn	Mw	Mz	Mz+1	Mv	PD
Peak 1	88139	52841	134050	265997	415393	245241	2.537

Figure S33. the GPC of Co(II) complex **4b** catalyzed polyisoprene in binary catalytic system (Table 2, entry 4).

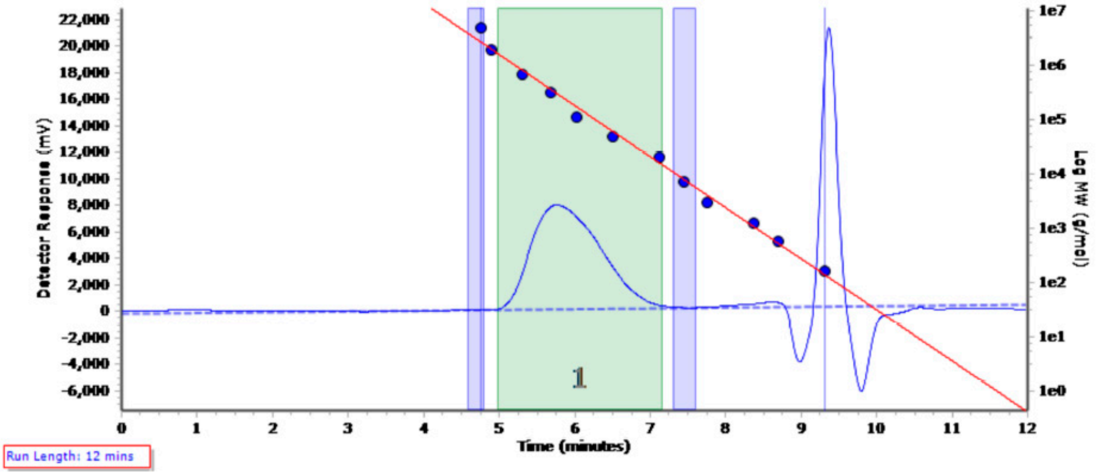

Molecular Weight Averages

Peak	Mp	Mn	Mw	Mz	Mz+1	Mv	PD
Peak 1	306524	139173	291365	477335	647813	451739	2.094

Figure S34. the GPC of Co(II) complex **5b** catalyzed polyisoprene in binary catalytic system (Table 2, entry 5).

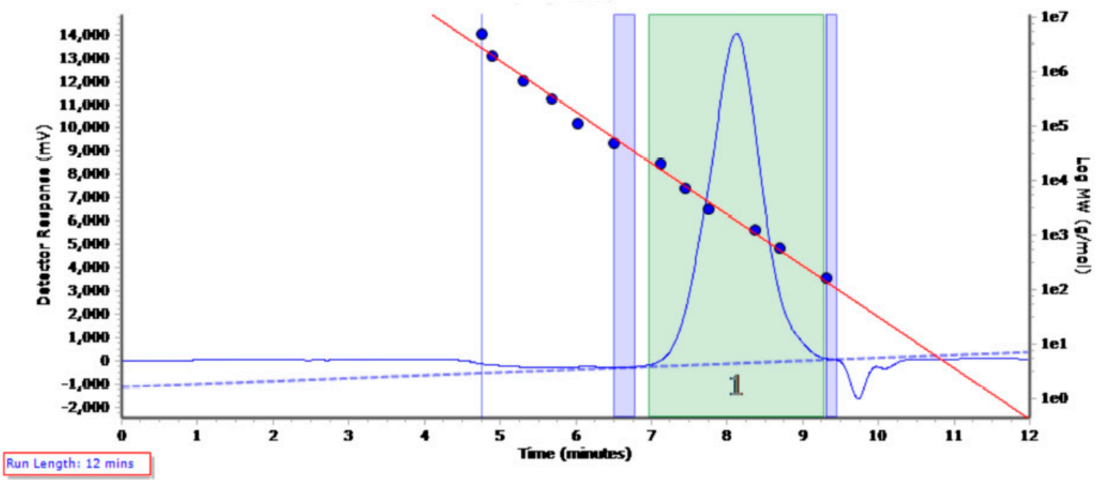

Molecular Weight Averages

Peak	Mp	Mn	Mw	Mz	Mz+1	Mv	PD
Peak 1	1847	1412	2513	4319	6907	4000	1.78

Figure S35. the GPC of Fe(II) complex **1a** catalyzed polyisoprene in ternary catalytic system (Table 3, entry 1).

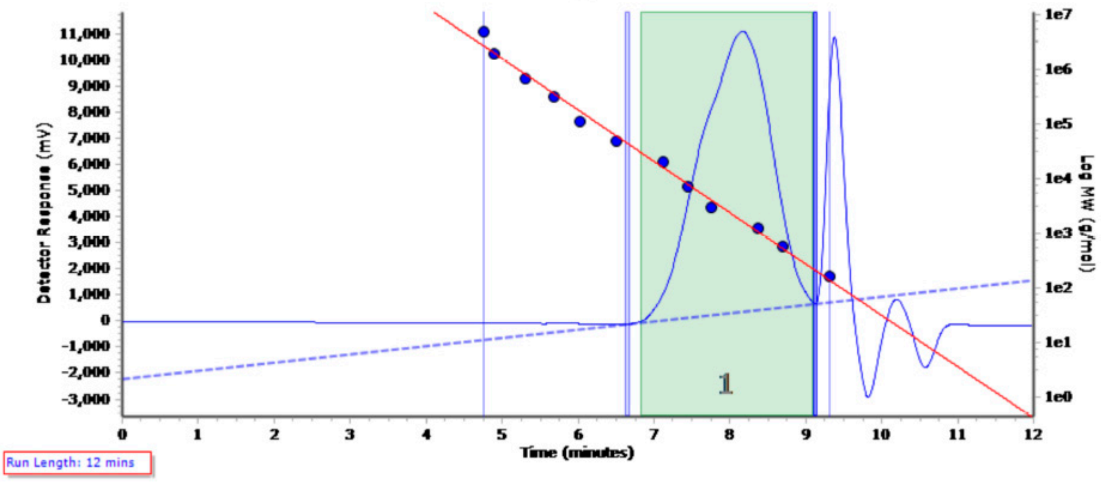

Molecular Weight Averages

Peak	Mp	Mn	Mw	Mz	Mz+1	Mv	PD
Peak 1	1687	1494	3189	6512	10676	5956	2.135

Figure S36. the GPC of Fe(II) complex **2a** catalyzed polyisoprene in ternary catalytic system (Table 3, entry 2).

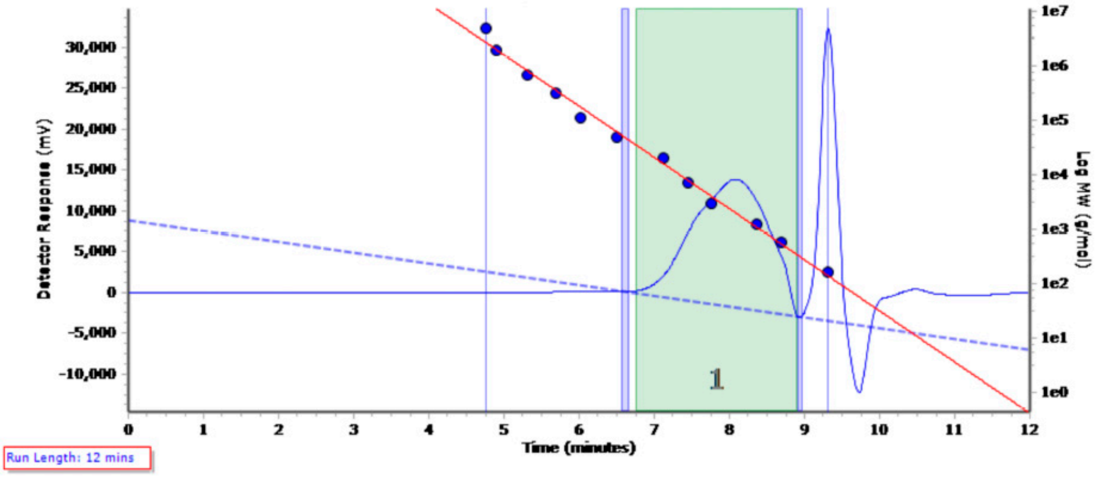

Molecular Weight Averages

Peak	Mp	Mn	Mw	Mz	Mz+1	Mv	PD
Peak 1	1949	1628	3741	8240	13843	7486	2.298

Figure S37. the GPC of Fe(II) complex **3a** catalyzed polyisoprene in ternary catalytic system (Table 3, entry 3).

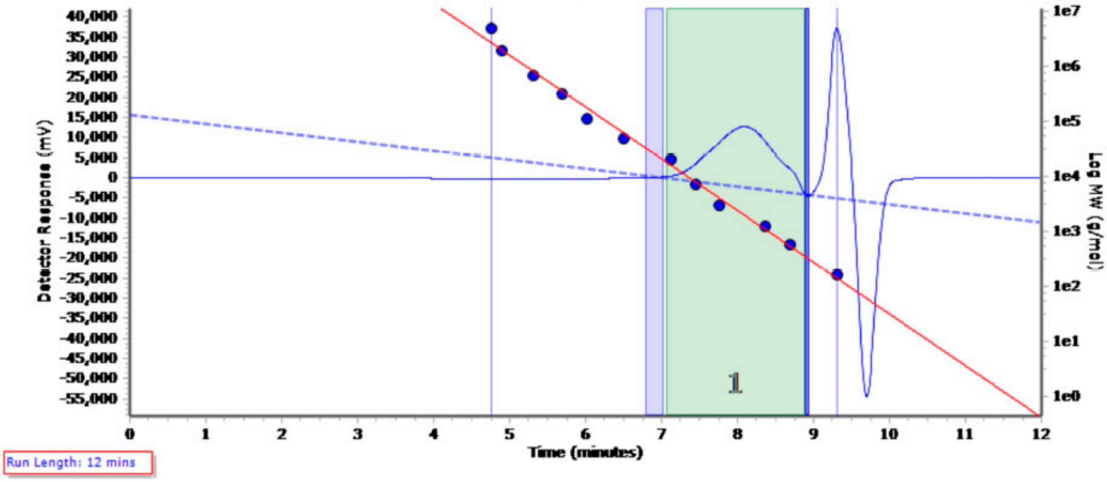

Molecular Weight Averages

Peak	Mp	Mn	Mw	Mz	Mz+1	Mv	PD
Peak 1	1880	1502	2815	5085	7737	4717	1.874

Figure S38. the GPC of Fe(II) complex **4a** catalyzed polyisoprene in ternary catalytic system (Table 3, entry 4).

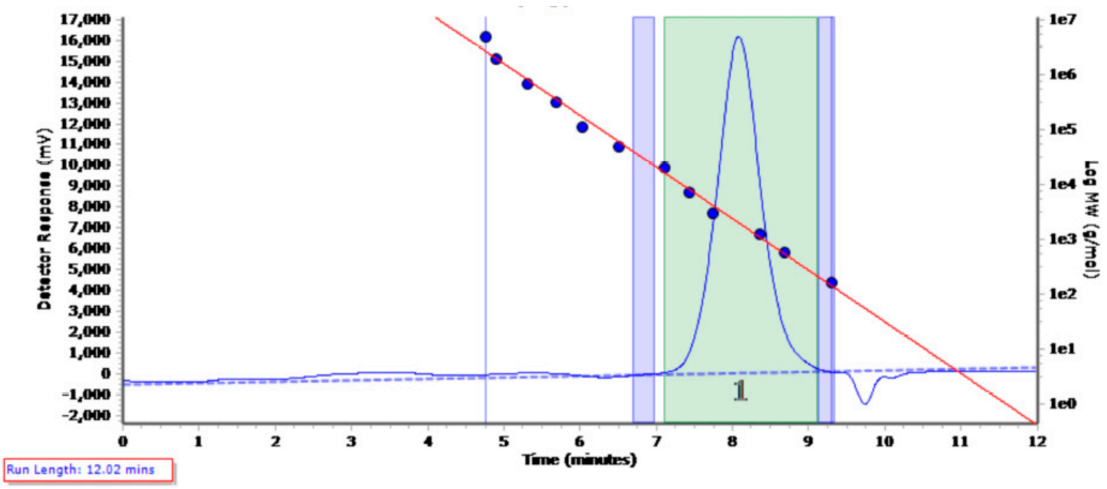

Molecular Weight Averages

Peak	Mp	Mn	Mw	Mz	Mz+1	Mv	PD
Peak 1	2021	1565	2349	3426	4918	3241	1.501

Figure S39. the GPC of Co(II) complex **1b** catalyzed polyisoprene in ternary catalytic system (Table 3, entry 6).

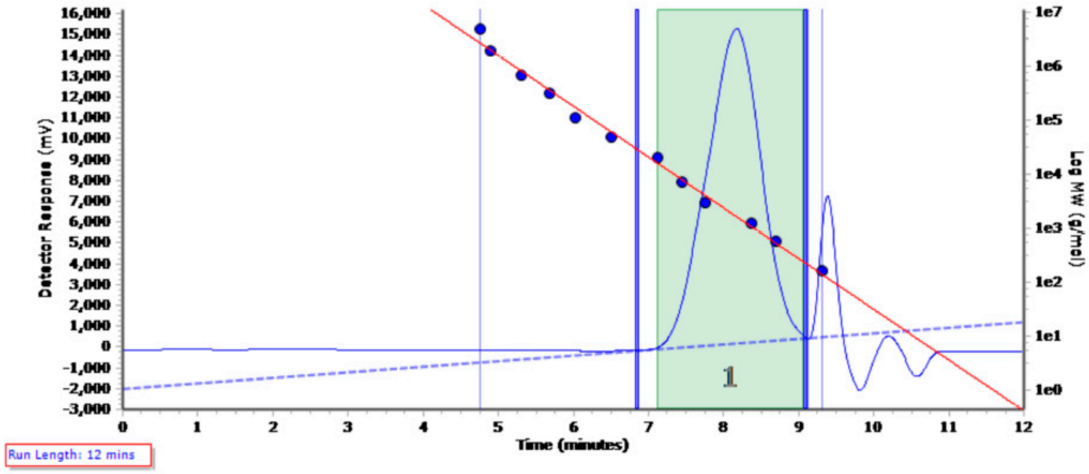

Molecular Weight Averages

Peak	Mp	Mn	Mw	Mz	Mz+1	Mv	PD
Peak 1	1657	1421	2352	3823	5650	3579	1.655

Figure S40. the GPC of Co(II) complex **2b** catalyzed polyisoprene in ternary catalytic system (Table 3, entry 7).

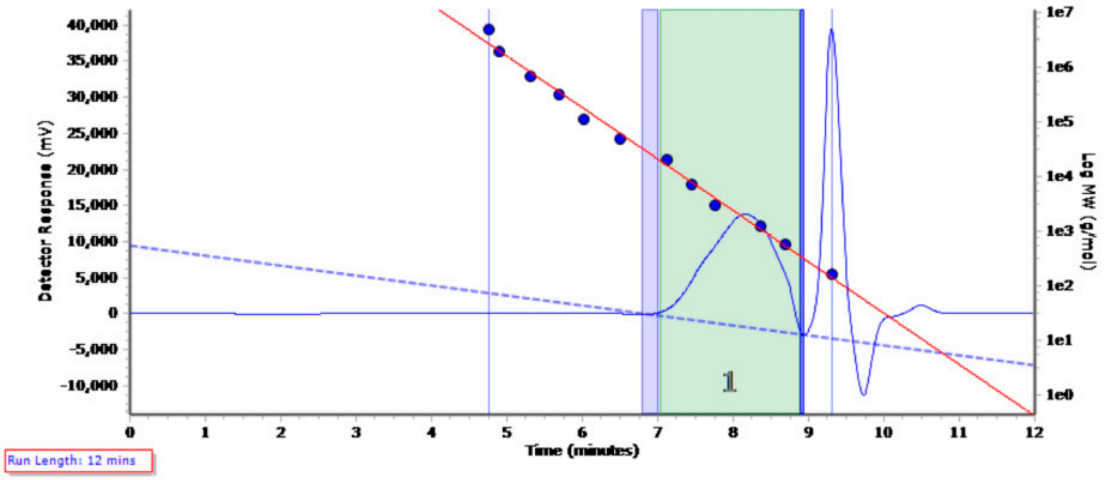

Molecular Weight Averages

Peak	Mp	Mn	Mw	Mz	Mz+1	Mv	PD
Peak 1	1598	1413	2793	5375	8308	4960	1.977

Figure S41. the GPC of Co(II) complex **3b** catalyzed polyisoprene in ternary catalytic system (Table 3, entry 8).

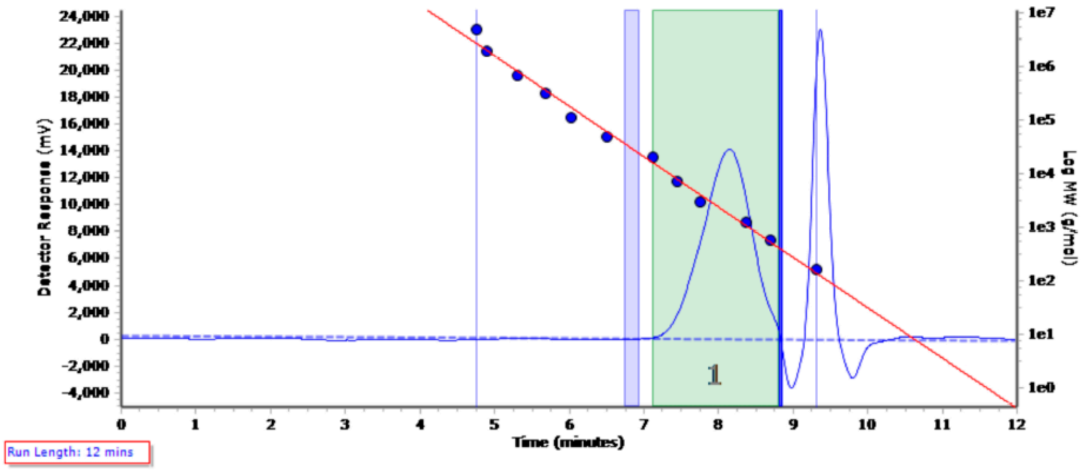

Molecular Weight Averages

Peak	Mp	Mn	Mw	Mz	Mz+1	Mv	PD
Peak 1	1749	1586	2431	3799	5602	3564	1.533

Figure S42. the GPC of Co(II) complex **4b** catalyzed polyisoprene in ternary catalytic system (Table 3, entry 9).

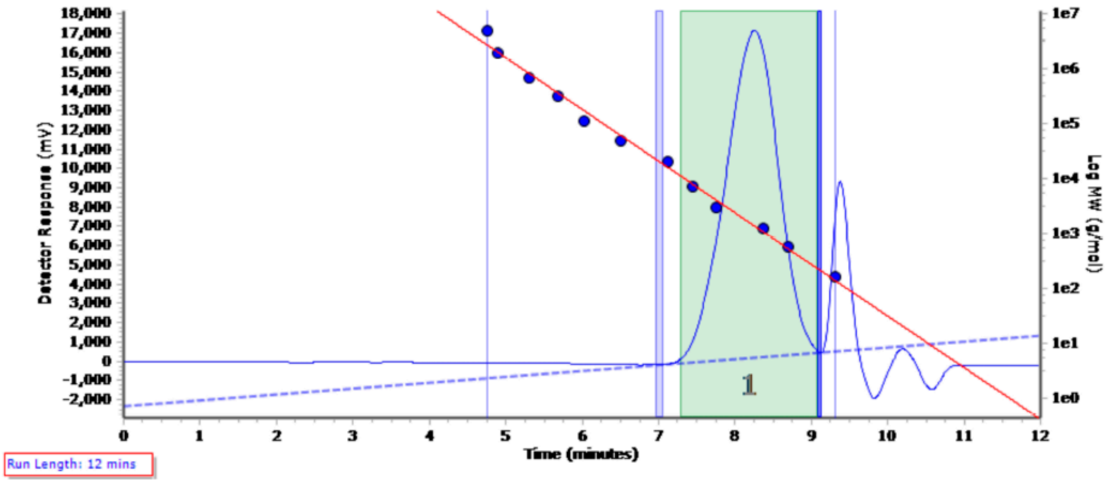

Molecular Weight Averages

Peak	Mp	Mn	Mw	Mz	Mz+1	Mv	PD
Peak 1	1383	1219	1915	2997	4325	2817	1.571

Figure S43. the GPC of Co(II) complex **5b** catalyzed polyisoprene in ternary catalytic system (Table 3, entry 10).

6. X-Ray Crystallography of Complexes

CCDC numbers of **3a**, **2b** and **3b** are 1830611, 1830608 and 1830612 respectively. These data can be obtained free of charge from The Cambridge Crystallographic Data Centre via www.ccdc.cam.ac.uk/data_request/cif.

Table S7. Crystal data and structure refinement for **3a, 2b and 3b.**

Identification Code	3a	2b	3b
Empirical formula	C30 H23 Cl2 F6 Fe N7	C58 H48 Cl12 Co4 F8 N12	C25 H16 Cl6 Co2 F6 N4
Formula weight	722.3	1726.2	816.98
Temperature	298(2) K	298(2) K	298(2) K
Wavelength	0.71073 A	0.71073 A	0.71073 A
Crystal system, space group	Triclinic, P-1	Triclinic, P-1	Triclinic, P-1
Unit cell dimensions	a = 11.7125(11) A alpha = 85.859(3) deg.<br>b = 12.1300(12) A beta = 77.814(2) deg.<br>c = 13.4818(13) A gamma = 63.5880(10) deg.	a = 13.7313(12) A alpha = 99.100(2) deg<br>b = 14.0523(13) A beta = 98.4230(10) deg.<br>c = 19.5067(16) A gamma = 97.8040(10) deg	a = 10.2435(9) A alpha = 94.2600(10) deg.<br>b = 12.5695(11) A beta = 108.994(2) deg.<br>c = 14.1690(12) A gamma = 95.6760(10) deg
Volume	1676.3(3) A^3	3627.5(5) A^3	1705.5(3) A^3
Z, Calculated density	2, 1.431 Mg/m^3	2, 1.580 Mg/m^3	2, 1.591 Mg/m^3
Absorption coefficient	0.674 mm^−1	1.407 mm^−1	1.498 mm^−1
F (000)	732	1728	808
Crystal size	0.43 x 0.30 x 0.20 mm	0.38 x 0.30 x 0.12 mm	0.23 x 0.20 x 0.15 mm
Theta range for data collection	2.40 to 25.02 deg.	2.22 to 25.02 deg.	2.51 to 25.02 deg.
Limiting indices	−13<=h<=13, −14<=k<=14, −15<=l<=16	−16<=h<=16, −16<=k<=9, −23<=l<=23	−12<=h<=12, −12<=k<=14, −16<=l<=12
Reflections collected/unique	5722 / 5722 [R(int) = 0.0000]	18531 / 12612 [R(int) = 0.0651]	8491 / 5881 [R(int) = 0.0324]

Completeness to theta = 25.02	97.00%	98.40%	97.80%
Absorption correction	Semi-empirical from equivalents	Semi-empirical from equivalents	Semi-empirical from equivalents
Max. and min. transmission	0.8769 and 0.7603	0.8493 and 0.6169	0.8065 and 0.7245
Refinement method	Full-matrix least-squares on F^2	Full-matrix least-squares on F^2	Full-matrix least-squares on F^2
Data/restraints/parameters	5722 / 0 / 419	12612 / 0 / 851	5881 / 1 / 419
Goodness-of-fit on F^2	1.106	1.276	1.022
Final R indices [I>2sigma(I)]	R1 = 0.0673, wR2 = 0.2077	R1 = 0.1219, wR2 = 0.3668	R1 = 0.0596, wR2 = 0.1533
R indices (all data)	R1 = 0.0934, wR2 = 0.2243	R1 = 0.1803, wR2 = 0.3955	R1 = 0.1137, wR2 = 0.1817
Largest diff. peak and hole	0.570 and −0.593 e.A^−3	1.821 and −1.919 e.A^−3	0.924 and −0.687 e.A^−3

Table S8. bond lengths and bong angles of Fe(II) complex **3a**.

**Bond lengths [Å]**			
Atom	length /Å	Atom	length /Å
Fe(1)-N(1)	2.204(5)	C(9)-C(10)	1.357(10)
Fe(1)-N(3)	2.215(5)	C(9)-H(9)	0.9300
Fe(1)-N(2)	2.304(5)	C(10)-C(11)	1.384(10)
Fe(1)-N(4)	2.322(5)	C(11)-C(12)	1.367(9)
Fe(1)-Cl(2)	2.3958(19)	C(11)-H(11)	0.9300
Fe(1)-Cl(1)	2.4207(18)	C(13)-C(14)	1.464(8)
F(1)-C(8)	1.353(7)	C(13)-H(13)	0.9300
F(2)-C(10)	1.380(8)	C(14)-C(15)	1.396(8)
F(3)-C(12)	1.359(7)	C(15)-C(16)	1.375(10)
F(4)-C(20)	1.359(7)	C(15)-H(15)	0.9300
F(5)-C(22)	1.362(7)	C(16)-C(17)	1.372(10)
F(6)-C(24)	1.372(8)	C(16)-H(16)	0.9300
N(1)-C(2)	1.343(7)	C(17)-C(18)	1.404(9)
N(1)-C(6)	1.354(7)	C(17)-H(17)	0.9300
N(2)-C(1)	1.285(7)	C(18)-H(18)	0.9300
N(2)-C(7)	1.423(7)	C(19)-C(20)	1.384(9)
N(3)-C(18)	1.344(7)	C(19)-C(24)	1.387(9)
N(3)-C(14)	1.350(7)	C(20)-C(21)	1.377(9)
N(4)-C(13)	1.275(7)	C(21)-C(22)	1.385(10)
N(4)-C(19)	1.432(7)	C(21)-H(21)	0.9300
N(5)-C(25)	1.116(11)	C(22)-C(23)	1.353(10)
N(6)-C(27)	1.117(12)	C(23)-C(24)	1.377(9)
N(7)-C(29)	1.158(19)	C(23)-H(23)	0.9300
C(1)-C(2)	1.462(8)	C(25)-C(26)	1.455(15)
C(1)-H(1)	0.9300	C(26)-H(26A)	0.9600
C(2)-C(3)	1.387(8)	C(26)-H(26B)	0.9600
C(3)-C(4)	1.385(9)	C(26)-H(26C)	0.9600
C(3)-H(3)	0.9300	C(27)-C(28)	1.469(14)
C(4)-C(5)	1.368(10)	C(28)-H(28A)	0.9600
C(4)-H(4)	0.9300	C(28)-H(28B)	0.9600
C(5)-C(6)	1.406(9)	C(28)-H(28C)	0.9600
C(5)-H(5)	0.9300	C(29)-C(30)	1.45(2)
C(6)-H(6)	0.9300	C(30)-H(30A)	0.9600
C(7)-C(8)	1.402(8)	C(30)-H(30B)	0.9600
C(7)-C(12)	1.404(8)	C(30)-H(30C)	0.9600
C(8)-C(9)	1.377(9)		
**Bond angles [deg]**			
Atom	Angle/°	Atom	Angle/°
N(1)-Fe(1)-N(3)	153.94(17)	F(3)-C(12)-C(11)	118.5(6)
N(1)-Fe(1)-N(2)	72.68(17)	F(3)-C(12)-C(7)	117.3(5)
N(3)-Fe(1)-N(2)	92.42(17)	C(11)-C(12)-C(7)	124.2(6)

N(1)-Fe(1)-N(4)	85.73(17)	N(4)-C(13)-C(14)	119.2(5)
N(3)-Fe(1)-N(4)	72.58(17)	N(4)-C(13)-H(13)	120.4
N(2)-Fe(1)-N(4)	89.53(17)	C(14)-C(13)-H(13)	120.4
N(1)-Fe(1)-Cl(2)	94.87(13)	N(3)-C(14)-C(15)	122.1(6)
N(3)-Fe(1)-Cl(2)	99.69(13)	N(3)-C(14)-C(13)	116.3(5)
N(2)-Fe(1)-Cl(2)	167.46(1	C(15)-C(14)-C(13)	121.6(6)
N(4)-Fe(1)-Cl(2)	91.02(13)	C(16)-C(15)-C(14)	119.3(6)
N(1)-Fe(1)-Cl(1)	105.26(13)	C(16)-C(15)-H(15)	120.3
N(3)-Fe(1)-Cl(1)	93.95(13)	C(14)-C(15)-H(15)	120.3
N(2)-Fe(1)-Cl(1)	84.03(12)	C(17)-C(16)-C(15)	118.8(6)
N(4)-Fe(1)-Cl(1)	164.85(13)	C(17)-C(16)-H(16)	120.6
Cl(2)-Fe(1)-Cl(1)	98.24(7)	C(15)-C(16)-H(16)	120.6
C(2)-N(1)-C(6)	117.4(5)	C(16)-C(17)-C(18)	119.8(6)
C(2)-N(1)-Fe(1)	117.5(4)	C(16)-C(17)-H(17)	120.1
C(6)-N(1)-Fe(1)	124.8(4)	C(18)-C(17)-H(17)	120.1
C(1)-N(2)-C(7)	118.8(5)	N(3)-C(18)-C(17)	121.4(6)
C(1)-N(2)-Fe(1)	114.3(4)	N(3)-C(18)-H(18)	119.3
C(7)-N(2)-Fe(1)	125.3(3)	C(17)-C(18)-H(18)	119.3
C(18)-N(3)-C(14)	118.5(5)	C(20)-C(19)-C(24)	116.1(5)
C(18)-N(3)-Fe(1)	124.4(4)	C(20)-C(19)-N(4)	120.9(5)
C(14)-N(3)-Fe(1)	117.0(4)	C(24)-C(19)-N(4)	122.7(6)
C(13)-N(4)-C(1)	119.1(5)	F(4)-C(20)-C(21)	118.7(6)
C(13)-N(4)-Fe(1)	114.5(4)	F(4)-C(20)-C(19)	117.8(5)
C(19)-N(4)-Fe(1)	126.0(4)	C(21)-C(20)-C(19)	123.5(6)
N(2)-C(1)-C(2)	118.8(5)	C(20)-C(21)-C(22)	116.9(6)
N(2)-C(1)-H(1)	120.6	C(20)-C(21)-H(21)	121.5
C(2)-C(1)-H(1)	120.6	C(22)-C(21)-H(21)	121.5
N(1)-C(2)-C(3)	122.8(5)	C(23)-C(22)-F(5)	119.3(6)
N(1)-C(2)-C(1)	115.7(5)	C(23)-C(22)-C(21)	122.4(6)
C(3)-C(2)-C(1)	121.5(5)	F(5)-C(22)-C(21)	118.3(6)
C(4)-C(3)-C(2)	119.5(6)	C(22)-C(23)-C(24)	118.7(6)
C(4)-C(3)-H(3)	120.2	C(22)-C(23)-H(23)	120.7
C(2)-C(3)-H(3)	120.2	C(24)-C(23)-H(23)	120.7
C(5)-C(4)-C(3)	118.7(6)	F(6)-C(24)-C(23)	119.0(6)
C(5)-C(4)-H(4)	120.6	F(6)-C(24)-C(19)	118.7(5)
C(3)-C(4)-H(4)	120.6	C(23)-C(24)-C(19)	122.4(6)
C(4)-C(5)-C(6)	119.1(6)	N(5)-C(25)-C(26)	179.1(12)
C(4)-C(5)-H(5)	120.4	C(25)-C(26)-H(26A)	109.5
C(6)-C(5)-H(5)	120.4	C(25)-C(26)-H(26B)	109.5
N(1)-C(6)-C(5)	122.4(6)	H(26A)-C(26)-H(26B)	109.5
N(1)-C(6)-H(6)	118.8	C(25)-C(26)-H(26C)	109.5
C(5)-C(6)-H(6)	118.8	H(26A)-C(26)-H(26C)	109.5
C(8)-C(7)-C(12)	114.7(5)	H(26B)-C(26)-H(26C)	109.5
C(8)-C(7)-N(2)	120.0(5)	N(6)-C(27)-C(28)	179.8(11)
C(12)-C(7)-N(2)	125.1(5)	C(27)-C(28)-H(28A)	109.5
F(1)-C(8)-C(9)	119.3(6)	C(27)-C(28)-H(28B)	109.5
F(1)-C(8)-C(7)	117.4(5)	H(28A)-C(28)-H(28B)	109.5
C(9)-C(8)-C(7)	123.2(6)	C(27)-C(28)-H(28C)	109.5
C(10)-C(9)-C(8)	117.8(6)	H(28A)-C(28)-H(28C)	109.5
C(10)-C(9)-H(9)	121.1	H(28B)-C(28)-H(28C)	109.5
C(8)-C(9)-H(9)	121.1	N(7)-C(29)-C(30)	178.5(17)
C(9)-C(10)-F(2)	118.6(7)	C(29)-C(30)-H(30A)	109.5
C(9)-C(10)-C(11)	123.4(6)	C(29)-C(30)-H(30B)	109.5
F(2)-C(10)-C(11)	118.0(6)	H(30A)-C(30)-H(30B)	109.5
C(12)-C(11)-C(10)	116.6(6)	C(29)-C(30)-H(30C)	109.5
C(12)-C(11)-H(11)	121.7	H(30A)-C(30)-H(30C)	109.5
C(10)-C(11)-H(11)	121.7	H(30B)-C(30)-H(30C)	109.5

Table S9. bond lengths and bong angles of Co(II) complex **2b**.

Bond lengths [Å]			
Atom	length /Å	Atom	length /Å
Co(1)-N(3)	2.121(10)	C(3)-H(3)	0.9300
Co(1)-N(1)	2.128(10)	C(4)-C(5)	1.33(2)
Co(1)-N(2)	2.140(10)	C(4)-H(4)	0.9300
Co(1)-N(4)	2.171(9)	C(5)-C(6)	1.336(19)
Co(1)-Cl(1)	2.429(3)	C(5)-H(5)	0.9300
Co(1)-Cl(2)	2.442(3)	C(6)-H(6)	0.9300
Co(2)-N(5)	2.102(11)	C(7)-C(8)	1.372(18)
Co(2)-N(7)	2.120(10)	C(7)-C(12)	1.397(17)
Co(2)-N(6)	2.149(10)	C(8)-C(9)	1.34(2)
Co(2)-N(8)	2.178(10)	C(9)-C(10)	1.34(2)
Co(2)-Cl(2)	2.435(3)	C(9)-H(9)	0.9300
Co(2)-Cl(1)	2.453(3)	C(10)-C(11)	1.39(2)
Co(3)-N(9)	2.053(13)	C(10)-H(10)	0.9300
Co(3)-Cl(5)	2.234(4)	C(11)-C(12)	1.366(18)
Co(3)-Cl(3)	2.240(4)	C(11)-H(11)	0.9300
Co(3)-Cl(4)	2.246(4)	C(13)-C(14)	1.459(18)
Co(4)-N(10)	2.031(13)	C(13)-H(13)	0.9300
Co(4)-Cl(8)	2.219(5)	C(14)-C(15)	1.363(17)
Co(4)-Cl(6)	2.237(4)	C(15)-C(16)	1.374(19)
Co(4)-Cl(7)	2.254(5)	C(15)-H(15)	0.9300
Cl(9)-C(57)	1.73(4)	C(16)-C(17)	1.343(18)
Cl(10)-C(57)	1.72(3)	C(16)-H(16)	0.9300
Cl(11)-C(58)	1.73(3)	C(17)-C(18)	1.391(18)
Cl(12)-C(58)	1.71(3)	C(17)-H(17)	0.9300
F(1)-C(8)	1.349(16)	C(18)-H(18)	0.9300
F(2)-C(12)	1.317(15)	C(19)-C(20)	1.392(18)
F(3)-C(20)	1.366(15)	C(19)-C(24)	1.396(19)
F(4)-C(24)	1.328(17)	C(20)-C(21)	1.389(19)
F(5)-C(32)	1.331(19)	C(21)-C(22)	1.35(2)
F(6)-C(36)	1.327(17)	C(21)-H(21)	0.9300
F(7)-C(44)	1.335(18)	C(22)-C(23)	1.37(2)
F(8)-C(48)	1.287(17)	C(22)-H(22)	0.9300
N(1)-C(2)	1.350(15)	C(23)-C(24)	1.35(2)
N(1)-C(6)	1.359(15)	C(23)-H(23)	0.9300
N(2)-C(1)	1.295(15)	C(25)-C(26)	1.501(18)
N(2)-C(7)	1.446(14)	C(25)-H(25)	0.9300
N(3)-C(18)	1.334(15)	C(26)-C(27)	1.376(18)
N(3)-C(14)	1.348(15)	C(27)-C(28)	1.36(2)
N(4)-C(13)	1.272(15)	C(27)-H(27)	0.9300
N(4)-C(19)	1.454(15)	C(28)-C(29)	1.35(2)
N(5)-C(26)	1.322(16)	C(40)-C(41)	1.34(2)
N(5)-C(30)	1.349(16)	C(40)-H(40)	0.9300
N(6)-C(25)	1.282(16)	C(41)-C(42)	1.41(2)
N(6)-C(31)	1.417(17)	C(41)-H(41)	0.9300
N(7)-C(42)	1.333(17)	C(42)-H(42)	0.9300
N(7)-C(38)	1.336(16)	C(43)-C(48)	1.37(2)
N(8)-C(37)	1.274(16)	C(43)-C(44)	1.38(2)
N(8)-C(43)	1.410(16)	C(44)-C(45)	1.39(2)
N(9)-C(49)	1.115(18)	C(45)-C(46)	1.33(2)
N(10)-C(51)	1.150(18)	C(45)-H(45)	0.9300
N(11)-C(53)	1.12(2)	C(46)-C(47)	1.36(2)
N(12)-C(55)	1.14(3)	C(46)-H(46)	0.9300
C(1)-C(2)	1.442(17)	C(47)-C(48)	1.39(2)
C(1)-H(1)	0.9300	C(47)-H(47)	0.9300
C(2)-C(3)	1.396(17)	C(49)-C(50)	1.43(2)
C(3)-C(4)	1.422(19)	C(50)-H(50A)	0.9600
C(28)-H(28)	0.9300	C(50)-H(50B)	0.9600
C(29)-C(30)	1.38(2)	C(50)-H(50C)	0.9600
C(29)-H(29)	0.9300	C(51)-C(52)	1.42(2)
C(30)-H(30)	0.9300	C(52)-H(52A)	0.9600
C(31)-C(32)	1.36(2)	C(52)-H(52B)	0.9600

C(31)-C(36)	1.42(2)	C(52)-H(52C)	0.9600
C(32)-C(33)	1.35(2)	C(53)-C(54)	1.39(3)
C(33)-C(34)	1.34(2)	C(54)-H(54A)	0.9600
C(33)-H(33)	0.9300	C(54)-H(54B)	0.9600
C(34)-C(35)	1.48(2)	C(54)-H(54C)	0.9600
C(34)-H(34)	0.9300	C(55)-C(56)	1.45(4)
C(35)-C(36)	1.33(2)	C(56)-H(56A)	0.9600
C(35)-H(35)	0.9300	C(56)-H(56B)	0.9600
C(37)-C(38)	1.472(19)	C(56)-H(56C)	0.9600
C(37)-H(37)	0.9300	C(57)-H(57A)	0.9700
C(38)-C(39)	1.425(19)	C(57)-H(57B)	0.9700
C(39)-C(40)	1.35(2)	C(58)-H(58A)	0.9700
C(39)-H(39)	0.9300	C(58)-H(58B)	0.9700

Bond angles [deg]

Atom	Angle/°	Atom	Angle/°
N(3)-Co(1)-N(1)	164.0(4)	N(3)-C(18)-C(17)	121.4(13)
N(3)-Co(1)-N(2)	97.5(4)	N(3)-C(18)-H(18)	119.3
N(1)-Co(1)-N(2)	77.0(4)	C(17)-C(18)-H(18)	119.3
N(3)-Co(1)-N(4)	75.3(4)	C(20)-C(19)-C(24)	115.8(13)
N(1)-Co(1)-N(4)	90.6(4)	C(20)-C(19)-N(4)	119.2(11)
N(2)-Co(1)-N(4)	99.3(4)	C(24)-C(19)-N(4)	124.8(12)
N(3)-Co(1)-Cl(1)	97.9(3)	F(3)-C(20)-C(21)	120.1(13)
N(1)-Co(1)-Cl(1)	96.8(3)	F(3)-C(20)-C(19)	116.5(12)
N(2)-Co(1)-Cl(1)	87.0(3)	C(21)-C(20)-C(19)	123.4(13)
N(4)-Co(1)-Cl(1)	171.3(3)	C(22)-C(21)-C(20)	117.5(15)
N(3)-Co(1)-Cl(2)	91.3(3)	C(22)-C(21)-H(21)	121.3
N(1)-Co(1)-Cl(2)	95.8(3)	C(20)-C(21)-H(21)	121.3
N(2)-Co(1)-Cl(2)	169.8(3)	C(21)-C(22)-C(23)	120.9(15)
N(4)-Co(1)-Cl(2)	87.9(3)	C(21)-C(22)-H(22)	119.6
Cl(1)-Co(1)-Cl(2)	86.71(11)	C(23)-C(22)-H(22)	119.6
N(5)-Co(2)-N(7)	163.6(4)	C(24)-C(23)-C(22)	121.5(15)
N(5)-Co(2)-N(6)	77.1(4)	C(24)-C(23)-H(23)	119.2
N(7)-Co(2)-N(6)	96.8(4)	C(22)-C(23)-H(23)	119.2
N(5)-Co(2)-N(8)	89.4(4)	F(4)-C(24)-C(23)	122.9(14)
N(7)-Co(2)-N(8)	76.6(4)	F(4)-C(24)-C(19)	116.3(13)
N(6)-Co(2)-N(8)	100.3(4)	C(23)-C(24)-C(19)	120.8(14)
N(5)-Co(2)-Cl(2)	94.9(3)	N(6)-C(25)-C(26)	116.5(12)
N(7)-Co(2)-Cl(2)	99.8(3)	N(6)-C(25)-H(25)	121.7
N(6)-Co(2)-Cl(2)	85.6(3)	C(26)-C(25)-H(25)	121.7
N(8)-Co(2)-Cl(2)	173.4(3)	N(5)-C(26)-C(27)	122.6(13)
N(5)-Co(2)-Cl(1)	96.7(3)	N(5)-C(26)-C(25)	115.6(11)
N(7)-Co(2)-Cl(1)	91.5(3)	C(27)-C(26)-C(25)	121.8(13)
N(6)-Co(2)-Cl(1)	169.4(3)	C(28)-C(27)-C(26)	118.0(15)
N(8)-Co(2)-Cl(1)	88.2(3)	C(28)-C(27)-H(27)	121.0
Cl(2)-Co(2)-Cl(1)	86.32(11)	C(26)-C(27)-H(27)	121.0
N(9)-Co(3)-Cl(5)	109.6(4)	C(29)-C(28)-C(27)	121.1(15)
N(9)-Co(3)-Cl (3)	102.7(4)	C(29)-C(28)-H(28)	119.5
Cl(5)-Co(3)-Cl(3)	113.70(18)	C(27)-C(28)-H(28)	119.5
N(9)-Co(3)-Cl(4)	105.9(4)	C(28)-C(29)-C(30)	118.1(15)
Cl(5)-Co(3)-Cl(4)	111.92(17)	C(28)-C(29)-H(29)	121.0
Cl(3)-Co(3)-Cl(4)	112.29(16)	C(30)-C(29)-H(29)	121.0
N(10)-Co(4)-Cl(8)	107.1(5)	N(5)-C(30)-C(29)	121.8(14)
N(10)-Co(4)-Cl(6)	104.9(5)	N(5)-C(30)-H(30)	119.1
Cl(8)-Co(4)-Cl(6)	113.23(18)	C(29)-C(30)-H(30)	119.1
N(10)-Co(4)-Cl(7)	103.8(4)	C(32)-C(31)-N(6)	122.4(14)
Cl(8)-Co(4)-Cl(7)	111.2(2)	C(32)-C(31)-C(36)	117.8(15)
Cl(6)-Co(4)-Cl(7)	115.52(17)	N(6)-C(31)-C(36)	119.3(13)
Co(1)-Cl(1)-Co(2)	93.38(11)	F(5)-C(32)-C(33)	117.9(16)
Co(2)-Cl(2)-Co(1)	93.48(11)	F(5)-C(32)-C(31)	119.5(15)
C(2)-N(1)-C(6)	119.4(11)	C(33)-C(32)-C(31)	122.7(18)
C(2)-N(1)-Co(1)	113.5(8)	C(34)-C(33)-C(32)	120.6(18)
C(6)-N(1)-Co(1)	126.8(9)	C(34)-C(33)-H(33)	119.7
C(1)-N(2)-C(7)	118.8(10)	C(32)-C(33)-H(33)	119.7
C(1)-N(2)-Co(1)	114.8(8)	C(33)-C(34)-C(35)	119.4(17)

C(7)-N(2)-Co(1)	125.7(8)	C(33)-C(34)-H(34)	120.3
C(18)-N(3)-C(14)	118.4(11)	C(35)-C(34)-H(34)	120.3
C(18)-N(3)-Co(1)	126.2(9)	C(36)-C(35)-C(34)	117.7(17)
C(14)-N(3)-Co(1)	115.0(8)	C(36)-C(35)-H(35)	121.1
C(13)-N(4)-C(19)	120.2(11)	C(34)-C(35)-H(35)	121.1
C(13)-N(4)-Co(1)	114.9(8)	F(6)-C(36)-C(35)	120.8(15)
C(19)-N(4)-Co(1)	123.7(8)	F(6)-C(36)-C(31)	117.5(13)
C(26)-N(5)-C(30)	118.3(11)	C(35)-C(36)-C(31)	121.7(16)
C(26)-N(5)-Co(2)	115.5(9)	N(8)-C(37)-C(38)	116.9(12)
C(30)-N(5)-Co(2)	125.9(9)	N(8)-C(37)-H(37)	121.5
C(25)-N(6)-C(31)	118.3(11)	C(38)-C(37)-H(37)	121.5
C(25)-N(6)-Co(2)	114.8(9)	N(7)-C(38)-C(39)	121.1(13)
C(31)-N(6)-Co(2)	125.8(8)	N(7)-C(38)-C(37)	117.5(12)
C(42)-N(7)-C(38)	119.7(12)	C(39)-C(38)-C(37)	121.4(13)
C(42)-N(7)-Co(2)	126.6(9)	C(40)-C(39)-C(38)	119.1(14)
C(38)-N(7)-Co(2)	113.5(8)	C(40)-C(39)-H(39)	120.4
C(37)-N(8)-C(43)	119.6(12)	C(38)-C(39)-H(39)	120.4
C(37)-N(8)-Co(2)	114.2(9)	C(41)-C(40)-C(39)	118.5(15)
C(43)-N(8)-Co(2)	125.5(9)	C(41)-C(40)-H(40)	120.7
C(49)-N(9)-Co(3)	172.1(14)	C(39)-C(40)-H(40)	120.7
C(51)-N(10)-Co(4)	173.8(15)	C(40)-C(41)-C(42)	122.1(15)
N(2)-C(1)-C(2)	117.6(11)	C(40)-C(41)-H(41)	118.9
N(2)-C(1)-H(1)	121.2	C(42)-C(41)-H(41)	118.9
C(2)-C(1)-H(1)	121.2	N(7)-C(42)-C(41)	119.4(14)
N(1)-C(2)-C(3)	122.0(11)	N(7)-C(42)-H(42)	120.3
N(1)-C(2)-C(1)	116.9(11)	C(41)-C(42)-H(42)	120.3
C(3)-C(2)-C(1)	121.1(12)	C(48)-C(43)-C(44)	118.5(14)
C(2)-C(3)-C(4)	116.2(13)	C(48)-C(43)-N(8)	120.2(13)
C(2)-C(3)-H(3)	121.9	C(44)-C(43)-N(8)	120.6(14)
C(4)-C(3)-H(3)	121.9	F(7)-C(44)-C(43)	119.0(14)
C(5)-C(4)-C(3)	119.2(14)	F(7)-C(44)-C(45)	119.5(16)
C(5)-C(4)-H(4)	120.4	C(43)-C(44)-C(45)	121.5(17)
C(3)-C(4)-H(4)	120.4	C(46)-C(45)-C(44)	118.2(18)
C(4)-C(5)-C(6)	123.1(15)	C(46)-C(45)-H(45)	120.9
C(4)-C(5)-H(5)	118.5	C(44)-C(45)-H(45)	120.9
C(6)-C(5)-H(5)	118.5	C(45)-C(46)-C(47)	122.6(18)
C(5)-C(6)-N(1)	120.0(13)	C(45)-C(46)-H(46)	118.7
C(5)-C(6)-H(6)	120.0	C(47)-C(46)-H(46)	118.7
N(1)-C(6)-H(6)	120.0	C(46)-C(47)-C(48)	119.0(17)
C(8)-C(7)-C(12)	116.8(12)	C(46)-C(47)-H(47)	120.5
C(8)-C(7)-N(2)	124.5(11)	C(48)-C(47)-H(47)	120.5
C(12)-C(7)-N(2)	118.4(11)	F(8)-C(48)-C(43)	118.1(13)
C(9)-C(8)-F(1)	120.9(14)	F(8)-C(48)-C(47)	121.8(16)
C(9)-C(8)-C(7)	123.5(15)	C(43)-C(48)-C(47)	120.1(16)
F(1)-C(8)-C(7)	115.6(12)	N(9)-C(49)-C(50)	176.3(18)
C(8)-C(9)-C(10)	118.5(15)	C(49)-C(50)-H(50A)	109.5
C(8)-C(9)-H(9)	120.8	C(49)-C(50)-H(50B)	109.5
C(10)-C(9)-H(9)	120.8	H(50A)-C(50)-H(50B)	109.5
C(9)-C(10)-C(11)	122.1(14)	C(49)-C(50)-H(50C)	109.5
C(9)-C(10)-H(10)	119.0	H(50A)-C(50)-H(50C)	109.5
C(11)-C(10)-H(10)	119.0	H(50B)-C(50)-H(50C)	109.5
C(12)-C(11)-C(10)	118.2(14)	N(10)-C(51)-C(52)	175(2)
C(12)-C(11)-H(11)	120.9	C(51)-C(52)-H(52A)	109.5
C(10)-C(11)-H(11)	120.9	C(51)-C(52)-H(52B)	109.5
F(2)-C(12)-C(11)	120.5(13)	H(52A)-C(52)-H(52B)	109.5
F(2)-C(12)-C(7)	118.5(12)	C(51)-C(52)-H(52C)	109.5
C(11)-C(12)-C(7)	120.9(13)	H(52A)-C(52)-H(52C)	109.5
N(4)-C(13)-C(14)	117.4(12)	H(52B)-C(52)-H(52C)	109.5
N(4)-C(13)-H(13)	121.3	N(11)-C(53)-C(54)	180(3)
C(14)-C(13)-H(13)	121.3	N(12)-C(55)-C(56)	174(4)
N(3)-C(14)-C(15)	122.2(12)	C(55)-C(56)-H(56A)	109.5
N(3)-C(14)-C(13)	115.4(10)	C(55)-C(56)-H(56B)	109.5
C(15)-C(14)-C(13)	122.4(12)	H(56A)-C(56)-H(56B)	109.5
C(14)-C(15)-C(16)	118.6(13)	C(55)-C(56)-H(56C)	109.5
C(14)-C(15)-H(15)	120.7	H(56A)-C(56)-H(56C)	109.5
C(16)-C(15)-H(15)	120.7	H(56B)-C(56)-H(56C)	109.5

C(17)-C(16)-C(15)	120.2(13)	Cl(10)-C(57)-Cl(9)	109(3)
C(17)-C(16)-H(16)	119.9	Cl(10)-C(57)-H(57A)	109.9
C(15)-C(16)-H(16)	119.9	Cl(9)-C(57)-H(57A)	109.9
C(16)-C(17)-C(18)	119.1(13)	Cl(10)-C(57)-H(57B)	109.9
C(16)-C(17)-H(17)	120.4	Cl(9)-C(57)-H(57B)	109.9
C(18)-C(17)-H(17)	120.4	H(57A)-C(57)-H(57B)	108.3
C(53)-C(54)-H(54A)	109.5	Cl(12)-C(58)-Cl(11)	109.3(19)
C(53)-C(54)-H(54B)	109.5	Cl(12)-C(58)-H(58A)	109.8
H(54A)-C(54)-H(54B)	109.5	Cl(11)-C(58)-H(58A)	109.8
C(53)-C(54)-H(54C)	109.5	Cl(12)-C(58)-H(58B)	109.8
H(54A)-C(54)-H(54C)	109.5	Cl(11)-C(58)-H(58B)	109.8
H(54B)-C(54)-H(54C)	109.5	H(58A)-C(58)-H(58B)	108.3

Table S10. bond lengths and bong angles of Co(II) complex **3b**.

Bond lengths [Å]			
Atom	Length/ Å	Atom	Length/ Å
Co(1)-N(3)	2.110(5)	C(4)-H(4)	0.9300
Co(1)-N(1)	2.131(5)	C(5)-C(6)	1.397(9)
Co(1)-N(2)	2.159(5)	C(5)-H(5)	0.9300
Co(1)-N(4)	2.165(4)	C(6)-H(6)	0.9300
Co(1)-Cl(1)	2.4647(17)	C(7)-C(8)	1.360(9)
Co(1)-Cl(2)	2.4881(16)	C(7)-C(12)	1.390(9)
Co(2)-Cl(4)	2.209(2)	C(8)-C(9)	1.376(10)
Co(2)-Cl(3)	2.2302(18)	C(9)-C(10)	1.365(11)
Co(2)-Cl(2)	2.3235(18)	C(9)-H(9)	0.9300
Co(2)-Cl(1)	2.3289(16)	C(10)-C(11)	1.337(11)
Cl(5)-C(25)	1.65(6)	C(11)-C(12)	1.378(10)
Cl(6)-C(25)	1.66(5)	C(11)-H(11)	0.9300
Cl(7)-C(26)	1.76(5)	C(13)-C(14)	1.464(8)
Cl(8)-C(26)	1.77(7)	C(13)-H(13)	0.9300
Cl(9)-C(27)	1.75(15)	C(14)-C(15)	1.368(8)
Cl(10)-C(27)	1.72(13)	C(15)-C(16)	1.399(9)
Cl(11)-C(28)	1.82(8)	C(15)-H(15)	0.9300
Cl(12)-C(28)	1.81(8)	C(16)-C(17)	1.379(9)
F(1)-C(8)	1.362(7)	C(16)-H(16)	0.9300
F(2)-C(10)	1.369(9)	C(17)-C(18)	1.385(9)
F(3)-C(12)	1.362(8)	C(17)-H(17)	0.9300
F(4)-C(20)	1.370(7)	C(18)-H(18)	0.9300
F(5)-C(22)	1.361(8)	C(19)-C(24)	1.362(9)
F(6)-C(24)	1.359(7)	C(19)-C(20)	1.370(8)
N(1)-C(6)	1.339(8)	C(20)-C(21)	1.383(9)
N(1)-C(2)	1.359(8)	C(21)-C(22)	1.366(10)
N(2)-C(1)	1.296(8)	C(21)-H(21)	0.9300
N(2)-C(7)	1.410(8)	C(22)-C(23)	1.344(10)
N(3)-C(18)	1.332(7)	C(23)-C(24)	1.365(9)
N(3)-C(14)	1.357(7)	C(23)-H(23)	0.9300
N(4)-C(13)	1.267(7)	C(25)-H(25A)	0.9700
N(4)-C(19)	1.422(7)	C(25)-H(25B)	0.9700
C(1)-C(2)	1.449(9)	C(26)-H(26A)	0.9700
C(1)-H(1)	0.9300	C(26)-H(26B)	0.9700
C(2)-C(3)	1.378(8)	C(27)-H(27A)	0.9700
C(3)-C(4)	1.375(10)	C(27)-H(27B)	0.9700
C(3)-H(3)	0.9300	C(28)-H(28A)	0.9700
C(4)-C(5)	1.362(10)	C(28)-H(28B)	0.9700

Bond angles [deg]			
Atom	Angle/°	Atom	Angle/°
N(3)-Co(1)-N(1)	165.31(19)	C(10)-C(11)-H(11)	121.6
N(3)-Co(1)-N(2)	94.9(2)	C(12)-C(11)-H(11)	121.6
N(1)-Co(1)-N(2)	77.4(2)	F(3)-C(12)-C(11)	119.1(7)
N(3)-Co(1)-N(4)	76.76(18)	F(3)-C(12)-C(7)	117.4(7)
N(1)-Co(1)-N(4)	92.19(18)	C(11)-C(12)-C(7)	123.5(8)
N(2)-Co(1)-N(4)	100.34(18)	N(4)-C(13)-C(14)	119.1(5)

N(3)-Co(1)-Cl(1)	93.94(14)	N(4)-C(13)-H(13)	120.5
N(1)-Co(1)-Cl(1)	95.47(16)	C(14)-C(13)-H(13)	120.5
N(2)-Co(1)-Cl(1)	168.58(15)	N(3)-C(14)-C(15)	123.2(6)
N(4)-Co(1)-Cl(1)	88.75(13)	N(3)-C(14)-C(13)	114.8(5)
N(3)-Co(1)-Cl(2)	96.27(13)	C(15)-C(14)-C(13)	122.0(6)
N(1)-Co(1)-Cl(2)	95.44(13)	C(14)-C(15)-C(16)	119.3(6)
N(2)-Co(1)-Cl(2)	84.75(13)	C(14)-C(15)-H(15)	120.4
N(4)-Co(1)-Cl(2)	171.61(13)	C(16)-C(15)-H(15)	120.4
Cl(1)-Co(1)-Cl(2)	87.07(5)	C(17)-C(16)-C(15)	117.8(6)
Cl(4)-Co(2)-Cl(3)	112.60(9)	C(17)-C(16)-H(16)	121.1
Cl(4)-Co(2)-Cl(2)	111.47(8)	C(15)-C(16)-H(16)	121.1
Cl(3)-Co(2)-Cl(2)	113.66(8)	C(16)-C(17)-C(18)	119.3(6)
Cl(4)-Co(2)-Cl(1)	110.98(9)	C(16)-C(17)-H(17)	120.3
Cl(3)-Co(2)-Cl(1)	112.51(7)	C(18)-C(17)-H(17)	120.3
Cl(2)-Co(2)-Cl(1)	94.32(6)	N(3)-C(18)-C(17)	123.4(6)
Co(2)-Cl(1)-Co(1)	89.18(5)	N(3)-C(18)-H(18)	118.3
Co(2)-Cl(2)-Co(1)	88.74(6)	C(17)-C(18)-H(18)	118.3
C(6)-N(1)-C(2)	117.7(5)	C(24)-C(19)-C(20)	115.9(6)
C(6)-N(1)-Co(1)	127.8(5)	C(24)-C(19)-N(4)	122.6(6)
C(2)-N(1)-Co(1)	114.1(4)	C(20)-C(19)-N(4)	121.2(6)
C(1)-N(2)-C(7)	119.9(5)	F(4)-C(20)-C(19)	118.5(6)
C(1)-N(2)-Co(1)	112.7(5)	F(4)-C(20)-C(21)	117.7(6)
C(7)-N(2)-Co(1)	125.6(4)	C(19)-C(20)-C(21)	123.8(7)
C(18)-N(3)-C(14)	117.0(5)	C(22)-C(21)-C(20)	115.2(7)
C(18)-N(3)-Co(1)	128.1(4)	C(22)-C(21)-H(21)	122.4
C(14)-N(3)-Co(1)	115.0(4)	C(20)-C(21)-H(21)	122.4
C(13)-N(4)-C(19)	120.0(5)	C(23)-C(22)-F(5)	118.4(8)
C(13)-N(4)-Co(1)	114.0(4)	C(23)-C(22)-C(21)	124.6(7)
C(19)-N(4)-Co(1)	124.3(4)	F(5)-C(22)-C(21)	117.0(8)
N(2)-C(1)-C(2)	120.2(6)	C(22)-C(23)-C(24)	116.7(8)
N(2)-C(1)-H(1)	119.9	C(22)-C(23)-H(23)	121.6
C(2)-C(1)-H(1)	119.9	C(24)-C(23)-H(23)	121.6
N(1)-C(2)-C(3)	122.1(7)	F(6)-C(24)-C(19)	117.3(6)
N(1)-C(2)-C(1)	114.9(5)	F(6)-C(24)-C(23)	118.9(7)
C(3)-C(2)-C(1)	123.0(7)	C(19)-C(24)-C(23)	123.8(7)
C(4)-C(3)-C(2)	119.6(7)	Cl(5)-C(25)-Cl(6)	122(4)
C(4)-C(3)-H(3)	120.2	Cl(5)-C(25)-H(25A)	106.8
C(2)-C(3)-H(3)	120.2	Cl(6)-C(25)-H(25A)	106.8
C(5)-C(4)-C(3)	119.1(7)	Cl(5)-C(25)-H(25B)	106.8
C(5)-C(4)-H(4)	120.5	Cl(6)-C(25)-H(25B)	106.8
C(3)-C(4)-H(4)	120.5	H(25A)-C(25)-H(25B)	106.6
C(4)-C(5)-C(6)	119.3(7)	Cl(7)-C(26)-Cl(8)	133(3)
C(4)-C(5)-H(5)	120.3	Cl(7)-C(26)-H(26A)	104.1
C(6)-C(5)-H(5)	120.3	Cl(8)-C(26)-H(26A)	104.1
N(1)-C(6)-C(5)	122.2(7)	Cl(7)-C(26)-H(26B)	104.1
N(1)-C(6)-H(6)	118.9	Cl(8)-C(26)-H(26B)	104.1
C(5)-C(6)-H(6)	118.9	H(26A)-C(26)-H(26B)	105.5
C(8)-C(7)-C(12)	114.5(7)	Cl(10)-C(27)-Cl(9)	103(8)
C(8)-C(7)-N(2)	121.5(6)	Cl(10)-C(27)-H(27A)	111.2
C(12)-C(7)-N(2)	123.6(7)	Cl(9)-C(27)-H(27A)	111.2
C(7)-C(8)-F(1)	117.7(6)	Cl(10)-C(27)-H(27B)	111.2
C(7)-C(8)-C(9)	125.2(7)	Cl(9)-C(27)-H(27B)	111.2
F(1)-C(8)-C(9)	117.1(7)	H(27A)-C(27)-H(27B)	109.1
C(10)-C(9)-C(8)	115.4(8)	Cl(12)-C(28)-Cl(11)	126(4)
C(10)-C(9)-H(9)	122.3	Cl(12)-C(28)-H(28A)	105.8
C(8)-C(9)-H(9)	122.3	Cl(11)-C(28)-H(28A)	105.8
C(11)-C(10)-C(9)	124.5(8)	Cl(12)-C(28)-H(28B)	105.8
C(11)-C(10)-F(2)	118.1(8)	Cl(11)-C(28)-H(28B)	105.8
C(9)-C(10)-F(2)	117.3(8)	H(28A)-C(28)-H(28B)	106.2
C(10)-C(11)-C(12)	116.8(7)		

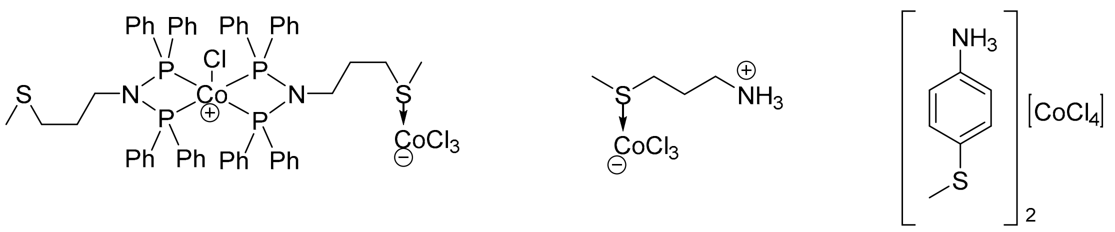

Figure S44. Three examples of structurally characterized cobalt complexes with anionic Co(II) centers presenting the [$CoCl_3$(RSR′)] and [$CoCl_4$] [(RSR′)]

7. Machanism of Formation of Polyisoprene.

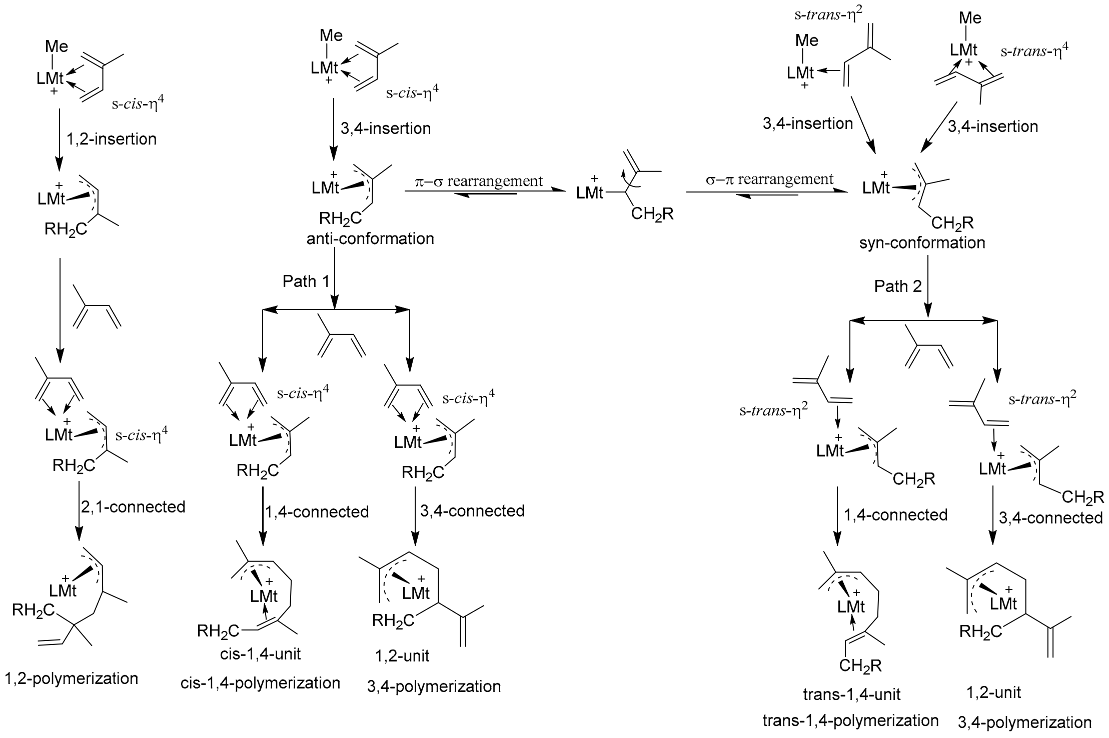

Figure S45. Machanism of Formation of Polyisoprene.
